# In Vitro Effects of Papaverine on Cell Proliferation, Reactive Oxygen Species, and Cell Cycle Progression in Cancer Cells

**DOI:** 10.3390/molecules26216388

**Published:** 2021-10-22

**Authors:** Daniella A. Gomes, Anna M. Joubert, Michelle H. Visagie

**Affiliations:** Department of Physiology, Faculty of Health Sciences, School of Medicine, University of Pretoria, Private Bag X323, Gezina, Pretoria 0031, South Africa; daniella.a.d.gomes@gmail.com (D.A.G.); annie.joubert@up.ac.za (A.M.J.)

**Keywords:** papaverine, cancer, morphology, proliferation, cell cycle

## Abstract

Papaverine (PPV) is an alkaloid isolated from the *Papaver somniferum*. Research has shown that PPV inhibits proliferation. However, several questions remain regarding the effects of PPV in tumorigenic cells. In this study, the influence of PPV was investigated on the proliferation (spectrophotometry), morphology (light microscopy), oxidative stress (fluorescent microscopy), and cell cycle progression (flow cytometry) in MDA-MB-231, A549, and DU145 cell lines. Exposure to 150 μM PPV resulted in time- and dose-dependent antiproliferative activity with reduced cell growth to 56%, 53%, and 64% in the MDA-MB-231, A549, and DU145 cell lines, respectively. Light microscopy revealed that PPV exposure increased cellular protrusions in MDA-MB-231 and A549 cells to 34% and 23%. Hydrogen peroxide production increased to 1.04-, 1.02-, and 1.44-fold in PPV-treated MDA-MB-231, A549, and DU145 cells, respectively, compared to cells propagated in growth medium. Furthermore, exposure to PPV resulted in an increase of cells in the sub-G_1_ phase by 46% and endoreduplication by 10% compared to cells propagated in growth medium that presented with 2.8% cells in the sub-G_1_ phase and less than 1% in endoreduplication. The results of this study contribute to understanding of effects of PPV on cancer cell lines.

## 1. Introduction

Cancer is one of the leading causes of death globally, with mortality rates increasing from 9.6 million in 2018 to 10 million in 2020 [1,2]. Simultaneously, the prevalence of cancer increased from 18.1 million to 19.3 million new cases, while death rates rose to approximately 19% in females and 43% in males [1,2]. It is projected that 28.4 million new cases will occur by 2040 should the rate trajectory remain consistent with the 2020 estimates [1]. Lung and breast cancer are the most common cancers globally, with lung cancer being the most common cause of cancer-related deaths, and breast cancer the fifth most common cause of cancer-related deaths. In 2020, approximately 2.2 million individuals were diagnosed with lung cancer, resulting in 1.8 million deaths [1]. In addition, 2.3 million individuals were diagnosed with breast cancer in 2020, resulting in 684,996 deaths [1]. Furthermore, approximately 1.4 million individuals were diagnosed with prostate cancer in 2020, resulting in 375,304 deaths [1].

The use of traditional plant-based medicine has been well documented and dates as far back as 2800 BC. Moreover, in today’s modern age, the use of plant-derived compounds has grown and is creating a separate industry focused on phytomedicine rather than synthetic compounds [3]. Phytomedicines account for approximately 60% of the total of anticancer agents currently in use [4]. Naturally occurring plant derived treatments have therefore become a large avenue of research to develop novel cancer treatment options with a higher therapeutic index [5,6,7,8].

Papaverine (PPV) is a naturally occurring non-narcotic alkaloid obtained from *Papaver somniferum*, commonly known as the opium poppy seed (poppies) [9]. Poppies have been used as an herbal medicine in Chinese and Indian medicine for their analgesic effects [4,9,10]. Despite being extracted from the poppy seed along with other opioids and alkaloids, the pharmacological activity of PPV does not possess any narcotic characteristics and is unrelated to the morphine classification of opioids, and it does not exert any analgesic effects [11,12]. PPV is approved by the Food and Drug Administration (FDA) of the United States of America as a vasodilator for the treatment of cerebral vasospasms and several coronary procedures, including subendocardial ischemia and erectile dysfunction [13,14,15,16,17]. The bioavailability of PPV is approximately 30% when taken orally [16,18,19,20]. Furthermore, these effects of PPV appear to be dose-dependent [19,21,22,23]. In addition, research studies have indicated that a 24-h and 48-h exposure to PPV at doses ranging from 0.01 to 1000 μM exhibited a dose-dependent cytotoxic effect in breast ductal-carcinoma (T47D), a triple negative breast carcinoma cell line, M.D. Anderson-Metastatic breast cancer (MDA-MB-231), an estrogen receptor positive breast carcinoma cell line, Michigan Cancer Foundation cell line 7 (MCF-7), colorectal carcinoma (HT 29), prostate carcinoma (PC-3), and fibrosarcoma (HT1080) cells. In addition, no cytotoxic effects were exhibited in non-tumorigenic human fibroblast (NHF) and mouse non-tumorigenic embryonic fibroblasts (NIH 3T3) T-cells at doses ranging from 0.01 to 1000 μM [4,9,24]. Cytotoxicity assays conducted on NIH 3T3 cells showed that exposure to high doses of PPV (100–1000 μM) reduced the percentage of cell growth to 90%. However, exposure to 0.01–1000 μM PPV in the tumorigenic cell lines, T47D, HT 29, and HT1080 resulted in a more prominent decrease in the percentage of cell growth to 20%, 30%, and 10%, respectively [9]. Additionally, research comparing the effects of PPV on PC-3 and NHF cells indicated that at a concentration of 200 μM PPV, cell viability was reduced to 10% and 90%, respectively [24]. This indicates that PPV may have a selective cytotoxicity towards tumorigenic cells while leaving non-tumorigenic cells either unaffected or less prominently affected [4,9,24].

Previous research has reported that PPV functions as a phosphodiesterase 10A (PDE10A) inhibitor which accounts for the anti-spasmodic effects observed in blood vessels when exposed to PPV [20,25]. Inhibition of PDE10A results in the increase in 3′,5′-cyclic adenosine monophosphate (cAMP) which has several downstream effects, including the alteration of the mitochondrial complex 1 [20,25,26,27]. Consequently, these effects may alter the production of reactive oxygen species (ROS) as the mitochondrial complex I is one of the main sources of ROS through phosphorylation by nicotinamide adenine dinucleotide hydrogen (NADH) [28]. It is therefore possible that the effects exerted by PPV may alter ROS production. Currently, there is limited research avalaible regarding the effects that PPV exerts on ROS production.

Previous studies have indicated that the naturally occurring compound, PPV, currently in clinical use for vasodilation purposes, might inhibit cell growth and potentially induces cell death in cancer cell lines. However, specific effects on biochemical pathways remain unclear. Therefore, this study investigated the effects of PPV on cell proliferation, morphology, oxidative stress, cell cycle progression, and cell death induction in a triple negative breast cancer cell line (MDA-MB-231), adenocarcinoma alveolar cancer cell line (A549), and a prostate cancer cell line (DU145). Although previous research has extensively explored the cytotoxic effects of PPV, little research has been conducted on the effects that PPV exerts in tumorigenic cell lines on morphology and oxidative stress whilst research of the effects of PPV on cell cycle progression has yielded contradicting results [4,24]. Therefore, in the present study, we conducted a cytotoxicity assay to determine the optimal dose range in the selected tumorigenic cell lines which were then implemented in further experimentation to establish the effects that PPV exerts on morphology, oxidative stress, and cell cycle progression, which can aid future anticancer studies. 

The data obtained in this study aided in the understanding of PPV’s antiproliferative influence on cancer cell lines. Furthermore, contributing to the existing knowledge regarding the influence of a naturally occurring compound in cancer cell lines will improve cancer researchers’ understanding of phytomedicinal compounds. The present study possibly provides new insight into the repurposing of non-addictive alkaloid, since it indicates that PPV exerts anti-proliferative activity, and induces oxidative stress and cell cycle abnormalities. The present study explores the drug repurposing of a natural vasodilator in cancer research, determining antiproliferative and anticancer effects to provide insights into the novel application of PPV, a non-addictive, non-narcotic alkaloid, which may have reduced side effects compared to current therapeutic cancer treatments. Understanding the phytomedicinal compounds and determining the benefits of these compounds in comparison to current synthetic treatments may help develop novel treatment options with reduced side effects and potentially improved survival. 

## 2. Results 

### 2.1. Cell Proliferation

#### Cell Number Determination Using Crystal Violet Staining (Spectrophotometry)

**Figure 1:**
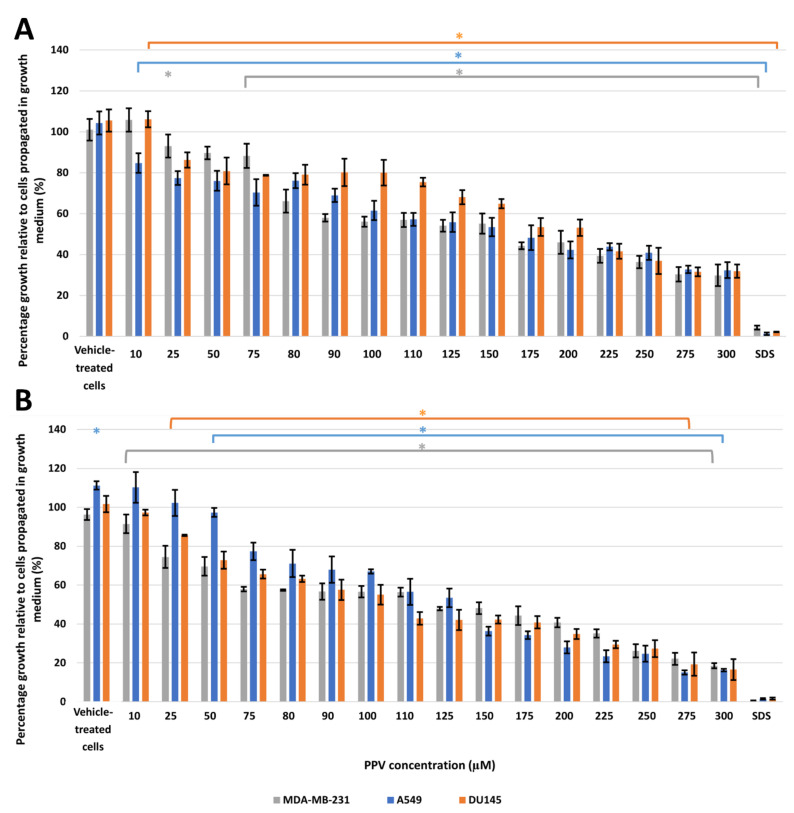
Spectrophotometry results of crystal violet staining demonstrating the effects of PPV (10–300 μM) on proliferation on MDA-MB-231, A549, and DU145 cell lines at 48 h (**A**) and 72 h (**B**). The average of three independent experiments is represented by the graph with error bars indicating standard deviation. The statistical significance is represented by an * when using the Student t-test with a *p* value of 0.05 compared to cells propagated in growth medium.

The crystal violet assay (spectrophotometry) results indicated that PPV exerted differential time- and concentration-dependent effects on cell growth in all three cell lines (Figure 1, Supplementary Materials 1). Exposure to PPV at 50, 100, 150, and 300 μM for 48 h in MDA-MB-231 cells resulted in a change in cell growth of 89%, 56%, 55% and 29%, respectively. In comparison, exposure to PPV at 50, 100, 150, and 300 μM for 48 h in A549 cells resulted in a change in cell growth of 76%, 61%, 53% and 32%, respectively, and exposure to PPV at 50, 100, 150, and 300 μM for 48 h in DU145 cells resulted in a change in cell growth of 80%, 80%, 64% and 31%, respectively (Figure 1). 

Exposure to PPV at 50, 100, 150, and 300 μM for 72 h in MDA-MB-231 cells resulted in a change in cell growth of 69%, 56%, 48%, and 18%, respectively. In comparison, exposure to PPV at 50, 100, 150, and 300, μM for 72 h in A549 cells resulted in a change in cell growth of 97.4%, 67.0%, 36.3%, and 16.3%, respectively, and exposure to PPV at 50, 100, 150, and 300 μM for 72 h in DU145 cells resulted in a change in cell growth of 72%, 55%, 42%, and 16%, respectively (Figure 1).

These results indicate that the PPV exerts differential time- and dose-dependent effects on cell proliferation in all three cell lines. Furthermore, the data demonstrates that PPV exerts optimal antiproliferative effects that are more prominently observed in the MDA-MB-231 and A549 cell lines after 48-h and 72-h exposure compared to the DU145 cell line. Thus, for all subsequent experiments, cell lines were exposed to PPV (10 μM, 50 μM, 100 μM, and 150 μM) for 48 h and 72 h to determine the effect of PPV on the morphology, H_2_O_2_ production, and cell cycle and cell death induction. 

### 2.2. Cell Morphology

#### Morphology Observation Using Light Microscopy

**Figure 2:**
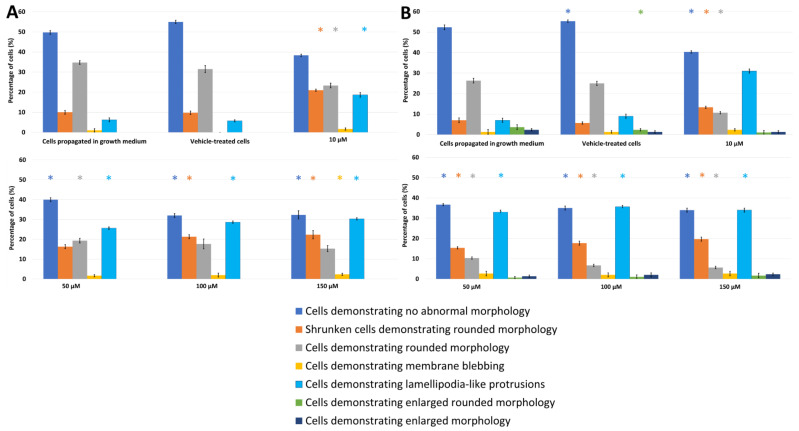
Light microscopy results demonstrating the effects of PPV (10–150 μM) on cell morphology on MDA-MB-231 cells at 48 h (**A**) and 72 h (**B**). Statistical significance is represented by an * when using the Student t-test with a *p* value of 0.05 compared to cells propagated in growth medium. Cells demonstrating enlarged and some cells exhibiting enlarged and rounded morphology were not observed after 48 h.

**Figure 3:**
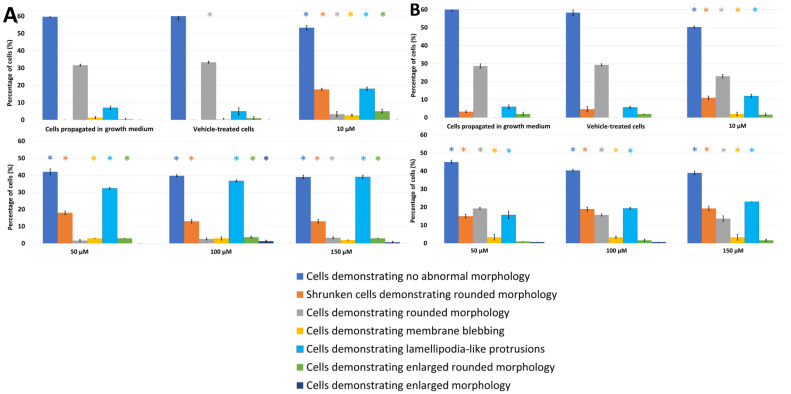
Light microscopy results demonstrating the effects of PPV (10–150 μM) on cell morphology on A549 cells at 48 h (**A**) and 72 h (**B**). Statistical significance is represented by an * when using the Student t-test with a *p* value of 0.05 compared to cells propagated in growth medium.

**Figure 4:**
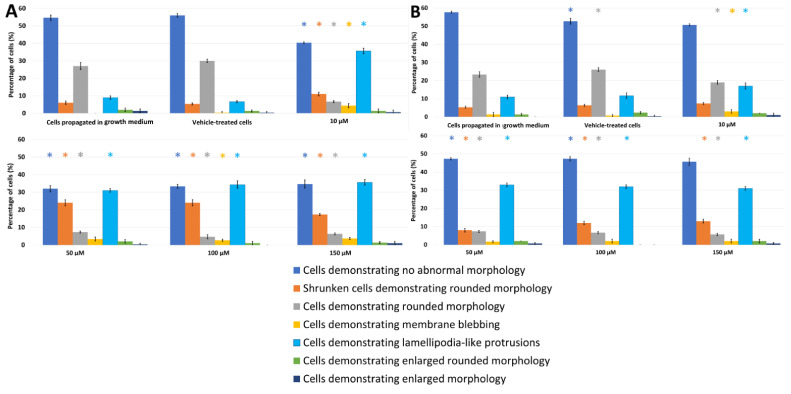
Light microscopy results demonstrating the effects of PPV (10–150 μM) on cell morphology on DU145 cells at 48 h (**A**) and 72 h (**B**). Statistical significance is represented by an * when using the Student t-test with a *p* value of 0.05 compared to cells propagated in growth medium.

The effects of PPV on cell morphology were investigated using light microscopy on MDA-MB-231, A549, and DU145 cell lines at 48 h and 72 h. This study was the first to demonstrate the effects of PPV in MDA-MB-231 and A549 cell lines. Light microscopy revealed that PPV decreased cell density and increased cell debris and abnormal morphological changes in a dose- and time-dependent manner in all three cell lines (Figure 2, Figure 3 and Figure 4, Supplementary Materials 2). Aberrant morphological observations after exposure to PPV included shrunken cells demonstrating rounded morphology, cells demonstrating rounded morphology, cells demonstrating membrane blebbing, cells demonstrating lamellipodia-like protrusions, and cells demonstrating enlarged rounded morphology and demonstrating enlarged morphology. Lamellipodia-like protrusions referring to cellular protrusions or extensions which act with the extracellular environment typically during cellular migration [29]. Cells demonstrating enlarged and some cells exhibiting enlarged and rounded morphology were only observed after 72 h in MDA-MB-231 cells. Furthermore, after exposure to 100 and 150 μM of PPV for 48 h and 72 h in A549 cells, enlarged rounded morphology and enlarged cells were observed.

**Figure 5:**
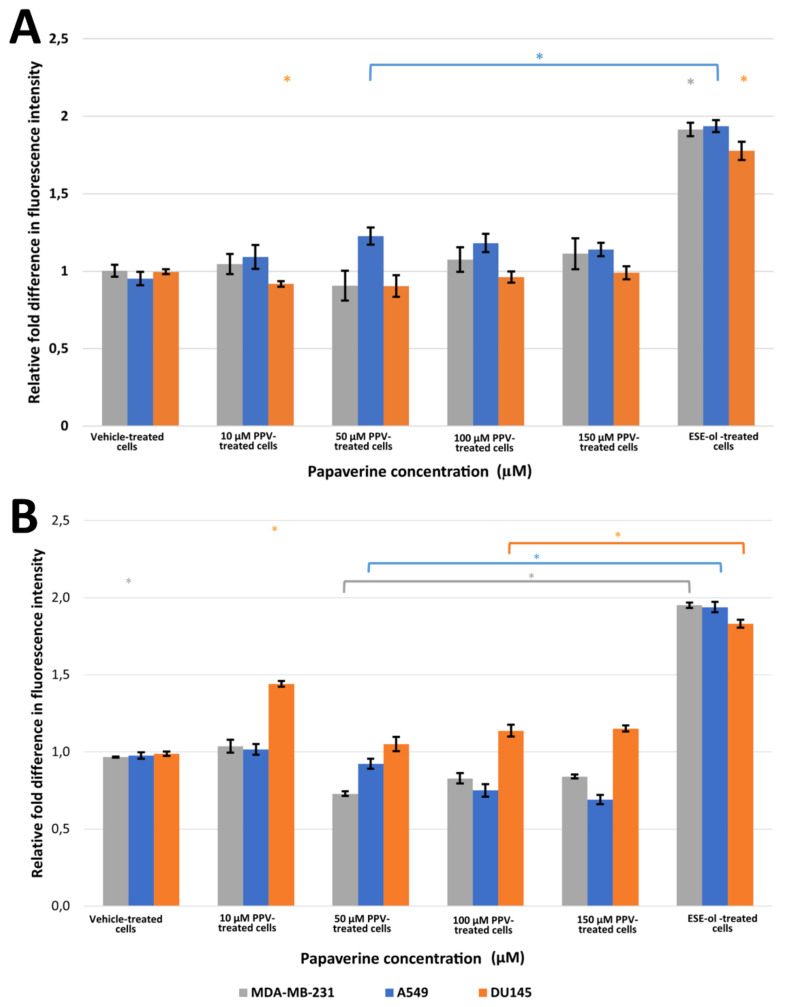
Fluorescence microscopy results of DCFDA staining demonstrating the effects of PPV (10–150 μM) on H_2_O_2_ production on MDA-MB-231, A549, and DU145 cell lines at 48 h (**A**) and 72 h (**B**). The average of three independent experiments is represented by the graph with error bars indicating standard deviation. Statistical significance is represented by an * when using the Student t-test with a *p* value of 0.05 compared to cells propagated in growth medium.

Exposure to PPV at 10, 50, 100, and 150 μM for 48 h in MDA-MB-231 cells resulted in significant aberrant morphological observations (Figure 2). Cells demonstrating lamellipodia-like protrusion abnormalities increased to 18%, 25%, 28%, and 30% in 10, 50, 100, and 150 μM, respectively. Exposure to PPV at 10, 50, 100, and 150 μM for 48 h in A549 cells resulted in significant aberrant morphological observations (Figure 3). Cells demonstrating lamellipodia-like protrusion abnormalities increased to 18%, 32%, 36%, and 39% in 10, 50, 100, and 150 μM, respectively, and exposure to PPV at 10, 50, 100, and 150 μM for 48 h in DU145 cells resulted in significant aberrant morphological observations (Figure 4). Cells demonstrating lamellipodia-like protrusion abnormalities increased to 35%, 31%, 34%, and 35% in 10, 50, 100, and 150 μM, respectively (Figure 5).

Exposure to PPV at 10, 50, 100, and 150 μM for 72 h in MDA-MB-231 cells resulted in significant aberrant morphological observations (Figure 2). Cells demonstrating lamellipodia-like protrusion abnormalities increased to 31%, 33%, 35%, and 34% in 10, 50, 100, and 150 μM, respectively. In comparison, exposure to PPV at 10, 50, 100, and 150 μM for 72 h in A549 cells resulted in significant aberrant morphological observations (Figure 3). Cells demonstrating lamellipodia-like protrusion abnormalities increased to 12%, 15%, 19%, and 23% in 10, 50, 100, and 150 μM, respectively, and exposure to PPV at 10, 50, 100, and 150 μM for 72 h in DU145 cells resulted in significant aberrant morphological observations (Figure 5). Cells demonstrating lamellipodia-like protrusion abnormalities increased to 17%, 33%, 32%, and 31% in 10, 50, 100, and 150 μM, respectively (Figure 4).

These data suggest that an increase in the concentration of PPV correlates with an increase in cells presenting with aberrant morphological manifestations such as lamellipodia-like protrusions. Furthermore, light microscopy confirmed the spectrophotometry results that PPV reduced cell growth in a time- and dose-dependent manner in MDA-MB-231, A549, and DU145 cell lines. Furthermore, studies have indicated that cells undergoing endoreduplication exhibit altered morphology including protrusions and enlarged cells [30]. It is therefore possible that some of the morphological abnormalities observed may be due to endoreduplication. However, further experimentation must be conducted to confirm these findings.

### 2.3. Oxidative Stress

#### Hydrogen Peroxide Production Using 2,7-Dichlorofluoresceindiacetate (DCFDA) (Fluorescent Microscopy)

The effects of PPV on hydrogen peroxide (H_2_O_2_) production was used as an indicator of oxidative stress. Exposure to PPV for 48 h resulted in a statistically significant increase in the fluorescent intensity in A549 cells when compared to cells propagated in growth medium (Figure 5, Supplementary Materials 3). A549 cells exposed to 10, 50, 100, and 150 μM of PPV for 48 h exhibited a fold increase to 1.09, 1.23, 1.18, and 1.14, respectively, relative to cells propagated in growth medium. However, MDA-MB-231 and DU145 cells exposed to PPV for 48 h exhibited no significant change in fluorescent intensity when compared to cells propagated in growth medium (Figure 5). Exposure to PPV for 72 h resulted in statistically significant decrease in fluorescent intensity in MDA-MB-231 and A549 cells when compared to cells propagated in growth medium (Figure 5). A549 cells exposed to 50, 100, and 150 μM of PPV for 72 h exhibited a fold decrease to 0.92, 0.75, and 0.69, respectively, relative to cells propagated in growth medium. MDA-MB-231 cells exposed to 50, 100, and 150 μM of PPV for 72 h exhibited a fold decrease to 0.73, 0.83, and 0.84 respectively relative to cells propagated in growth medium (Figure 5). A statistically significant fold increase to 1.44, 1.14, and 1.15 was observed in DU145 cells exposed to PPV for 72 h at a concentration of 10, 100, and 150 μM, respectively, relative to cells propagated in growth medium (Figure 5).

### 2.4. Cell Cycle Progression and Cell Death Induction

#### Cell Cycle Analysis Using Propidium Iodide Staining (Flow Cytometry)

Flow cytometry using propidium iodide staining and ethanol fixation allowed for the quantification of cell cycle distributions and cell death after exposure to PPV. The data obtained is the first to show the effects of PPV in A549 and DU145 cells on cell cycle progression. MDA-MB-231 cells exposed to 10, 50, 100, and 150 μM of PPV for 48 h exhibited a statistically significant increase of 4%, 8%, 10%, and 8% of cells occupying the sub-G_1_ phase, respectively, when compared to cells propagated in growth medium (Table 1). Similarly, MDA-MB-231 cells exposed to 10, 50, 100, and 150 μM of PPV for 72 h exhibited a statistically significant increase of 9%, 5%, 9%, and 46% of cells in the sub-G_1_ phase, respectively, compared to cells propagated in growth medium (Table 1, Supplementary Materials 4). 

Furthermore, a statistically significant increase of 1.09%, 1.06%, and 2.14% of cells in endoreduplication was observed when exposed to 10, 50, and 100 μM of PPV for 48 h, respectively, when compared to cells propagated in growth medium. Whilst a statistically significant increase of 3.4%, 10%, 10%, and 4.2% of cells in endoreduplication was observed when exposed to 10, 50, and 100 μM of PPV for 72 h, respectively, when compared to cells propagated in growth medium. 

A549 cells exposed to 10, 50, 100, and 150 μM of PPV for 48 h exhibited a statistically significant increase of 4%, 8%, 10%, and 8% of cells in the sub-G_1_ phase, respectively, when compared to cells propagated in growth medium. A549 cells exposed to 50, 100, and 150 μM of PPV for 72 h exhibited a statistically significant increase of 6%, 2%, and 6% of cells in the sub-G_1_ phase, respectively, when compared to cells propagated in growth medium (Table 2). Additionally, a statistically significant increase of 2.3% of cells in endoreduplication was observed when exposed to PPV for 48 h when compared to cells propagated in growth medium. Furthermore, a statistically significant increase of 3%, 8%, 2%, and 3% of cells in endoreduplication was observed when exposed to 10, 50, 100, and 150 μM of PPV for 72 h, respectively, when compared to cells propagated in growth medium.

DU145 cells exposed to 10, 50, 100, and 150 μM of PPV for 48 h exhibited a statistically significant increase of 9%, 7%, 4%, and 5% of cells in the sub-G_1_ phase, respectively, when compared to cells propagated in growth medium. DU145 cells exposed to 10, 50, 100, and 150 μM of PPV for 72 h exhibited a statistically significant increase of 13%, 10%, 14%, and 23% of cells in the sub-G_1_ phase, respectively, when compared to cells propagated in growth medium (Table 3). Furthermore, a statistically significant increase of 1.2%, 1.6%, and 0.8% of cells in endoreduplication was observed when exposed to 10, 50, and 100 μM of PPV for 48 h, respectively, when compared to cells propagated in growth medium, whilst a statistically significant increase of 3%, 9%, 4%, and 4% of cells in endoreduplication was observed when exposed to 10, 50, 100, and 150 μM of PPV for 72 h, respectively, when compared to cells propagated in growth medium. These results confirm the findings observed in the morphology studies, as it was suggested that the abnormal morphology observed in this study is indicative of endoreduplication. 

## 3. Discussion

Several studies have indicated that PPV exerts antiproliferative effects in tumorigenic cell lines while leaving non-tumorigenic cell lines less affected [4,9,24]. However, there is limited literature demonstrating the influence of PPV on other cellular phenomena and signal transduction in tumorigenic cell lines. Therefore, the effects of the benzylisoquinoline alkaloid, PPV, were investigated on cell proliferation, morphology, H_2_O_2_ production, and cell cycle progression in MDA-MB-231, A549, and DU145 cells. The proliferation study using crystal violet staining (10–300 μM) at 24 h, 48 h, 72 h, and 96 h was implemented as a time and dose study and revealed that PPV causes time- and dose-dependent cytotoxic effects in MDA-MB-231, A459, and DU145 cells. Previous studies have indicated that exposure to PPV for 48 h reduces cell viability with a half-maximal inhibitory concentration (IC50) of more than 10 μM in MDA-MB-231, MCF7, and PC-3 cells [4,24]. Furthermore, cytotoxicity assays using the 3-(4,5-dimethylthiazol-2-yl)-2,5-diphenyltetrazolium bromide (MTT) assay revealed that cell growth was reduced to 38%, 35%, 20%, and 15% in human hepatoma (HepG-2), HT 29, T47D, and HT 1080 cells after 48 h of exposure to PPV [9]. PPV exerted cytotoxic effects in tumorigenic cell lines, indicating that these effects of PPV were cell line-dependent [9,31]. These results are supported by the current study which indicated that PPV reduced cell viability in MDA-MB-231 and A549 cells more at 48 h and 72 h when compared to DU145 cells. 

Light microscopy revealed that at exposure times of 48 h and 72 h, all three cell lines were affected morphologically by PPV (10–150 μM) and indicated a reduction in cell density. An increase in aberrant morphological changes was observed that correlated with an increase in PPV concentration in all three cell lines, further supporting the suggestion that the effects exerted by PPV are dose-dependent. Additionally, a more prominent increase in abnormal morphology was present after exposure to PPV for 72 h, with more statistically significant morphological alterations seen in MDA-MB-231 cells compared to the A549 and DU145 cells. Previous research indicated that PPV exhibited no significant changes on the morphology of DU145 cells after 48 h [32]. Whilst these findings are supported by the present study, DU145 cells did exhibit more notable morphological alterations after 72 h. Furthermore, studies have indicated that cells undergoing endoreduplication exhibit aberrant morphological changes including enlarged morphology and in some cases, membrane projections; these alterations are similar to the morphological abnormalities observed in the present study [30].

A statistically significant increase in H_2_O_2_ production as an indicator of oxidative stress in A549 cells in comparison to MDA-MB-231 and DU145 cells was observed after 48 h exposure to PPV. Findings indicated that an increase in PPV concentration resulted in a decrease in fluorescent intensity in A549 cells; however, PPV does result in an increase in fluorescent intensity in comparison to cells propagated in growth medium only. Furthermore, exposure to PPV for 72 h indicated that an increase in PPV concentration resulted in a decrease in fluorescent intensity in all three cell lines. However, the fluorescent intensity in DU145 cells was higher than cells propagated in growth medium whilst the fluorescent intensity in MDA-MB-231 and A549 cells was lower than cells propagated in growth medium. 

Previous research has suggested that PPV inhibits PDE10A; consequently, the levels of available cAMP decrease [20,25,26,27]. Alterations to cAMP have been shown to affect the mitochondrial complex 1 which is the start point for the electron transport chain [28,33]. Therefore, the inhibitory effect PPV exerts on PDE10A may subsequently influence H_2_O_2_ production via the cAMP and mitochondrial complex 1 signalling cascade [20,25,26,27,28,33]. Prior research reported that mitochondrial respiration and the mitochondrial complex 1 has been affected by PPV, implicating the inhibition of PDE10A as a potential cause [17,20]. As indicated by the DCFDA staining, ROS production is affected by PPV. It is possible that these measurable affects are a result of the inhibition of PDE10A. However, further investigation must be conducted to confirm if these effects are connected. 

Cell cycle progression revealed that PPV induced a marked increase of cells in the sub-G_1_ peak, variable changes in the percentage of cells in the S and G_2_M phase, and a change in the percentage of cells in the endoreduplication phase when compared to cells propagated in growth medium at 48 h and 72 h. Endoreduplication has been described as a process by which cells that have undergone DNA damage continue to enter the cell cycle without dividing, resulting in polyploid cells [30]. This results in cells that can avoid programmed cell death. It has been suggested that when cells undergo endoreduplication, an initial period of inhibited cell proliferation occurs. However, subsequent to the initial cell growth inhibition, cells are still able to progress through the cell cycle and enter the S and G_2_M phase before undergoing endoreduplication [30]. Cells therefore have an increase in DNA and are ultimately larger in size. This leads to a peak in the cell cycle beyond the G_2_M peak [30]. Cell cycle progression after exposure to PPV showed an increase in cells undergoing endoreduplication when compared to cells propagated in growth medium and the vehicle-treated cells. The present study indicated that the effects exerted by PPV on cell cycle in all three cell lines are time- and dose-dependent. These results indicated that the effects are cell line specific, supporting previous studies [4,24].

The present study therefore contributes to the application of an existing natural vasodilator to cancer research and treatment by establishing its effects on proliferation, H_2_O_2_ production, and cell death induction. The results indicate time, dose, and cell line specific effects of PPV on cell proliferation, morphology, oxidative stress, and cell cycle progression. Developing an understanding of this natural herbal compound used in traditional and conventional medicine may aid in the development of novel phytomedicinal treatments which can potentially reduce the side effects observed in current treatments in cancer care and cancer research [4]. Understanding the compound’s cell line specificity may aid in its use as an antiproliferative agent; however, this must be further investigated. 

## 4. Materials and Methods

### 4.1. Materials

#### 4.1.1. Cell Lines

Triple negative breast cancer (TNBC) is a subtype of breast cancer that is highly invasive and characterised by the lack of estrogen receptors (ER), progesterone receptors (PR) and does not overproduce human epidermal growth factor receptor 2 (HER2) [34,35]. M.D. Anderson-Metastasis breast cancer-231 (MDA-MB-231) is a TNBC cell line that is highly invasive and tumorigenic with limited therapeutic targets. Previous research indicated cytotoxic effects of PPV on MDA-MB-231 cells with little insight into the effects on morphology, H_2_O_2_ production, and limited research on cell cycle progression [4]. Type II alveolar epithelium cells are found within the lungs, despite covering a small surface area of the alveolus; there are more type II alveolar epithelium cells than type I alveolar epithelium cells, as a result, type II alveolar epithelium adenocarcinomas are typically more common [36]. The A549 cell line is an alveolar adenocarcinoma cell line that exhibits type II alveolar cell characteristics, including larger pores to allow for increased diffusion [36]. Currently, there is limited research on the effects of PPV on A549 cells with studies focusing more on cytotoxicity and mitochondrial effects than morphology and cell cycle progression [17]. Human prostate adenocarcinoma (DU145) is a metastatic prostate adenocarcinoma cell line isolated from brain lesions in a 69-year-old male in 1975 [37]. Initial cultures of this cell line did not indicate any sensitivity to hormones as cells propagated in foetal calf serum (FCS) grew at the same rate as cells propagated in bull serum [37]. This cell line is an androgen receptor (AR) negative cell line that does not express prostate specific antigen (PSA) [37,38]. Currently, most research on the effects of PPV on prostate cancers has focused on PC-3 cells with few studies exploring the effects of PPV in DU145 cells [24,32]. 

MDA-MB-231, A549, and DU145 cells were obtained from the American Type Culture Collection (Manassas, Virginia, United States of America). Cells were maintained in Dulbecco’s Modified Eagle Growth medium (DMEM) containing 5 mM L-glutamine, 4 mM sodium pyruvate, 3 g/L glucose, 10% heat-inactivated FCS (56 °C, 30 min), 100 U/mL penicillin G, 100 mg/mL streptomycin and fungizone (250 mg/l) at 37 °C and 5% CO_2_ in a humidified atmosphere in 75-cm^2^ tissue flasks.

#### 4.1.2. Chemicals and Materials

All reagents and chemicals were purchased from Sigma Chemical Co. (St. Louis, MO, USA) and all plasticware were purchased from Lasec^®^ SA (Pty) Ltd. (Johannesburg, Gauteng) and supplied by Cellstar^®^, (Greiner, Germany) unless otherwise specified. PPV was purchased from Merck (Darmstadt, Germany) and was dissolved in dimethyl sulfoxide (DMSO) to a concentration of 50 mM. Appropriate controls were used including a negative control where cells were propagated in complete growth media and a vehicle-treated control (DMSO) where cells were exposed to equal amounts of the vehicle solvent solution as in PPV-treated cells, where the *v/v*% of DMSO did not exceed 0.35%.

### 4.2. Methods

#### 4.2.1. Cell Proliferation

##### Cell Number Determination Using Crystal Violet Staining (Spectrophotometry)

The crystal violet staining technique involves a powdered triphenylmethane cation dye which binds to the deoxyribonucleic acid (DNA) of proliferating cells allowing for the rapid quantification of proliferating cells in a monolayer [39]. The intensity of the colour of the dye correlates with cell numbers which will be quantified as absorbance by means of a spectrophotometer at a wavelength of 570 nm [40]. Therefore, the effects of PPV on cell viability were determined by crystal violet staining on MDA-MB-231, A549, and DU145 cell lines. 

Cells were seeded in a sterile 96-well culture plate at a cell density of 5000 cells per well prior to incubation at 37 °C and 5% CO_2_ in a humified atmosphere for 24 h to allow for cell attachment. Subsequently, cells were exposed to PPV (10–300 μm) for 24 h, 48 h, 72 h, or 96 h since previous studies have indicated optimal antiproliferative activity within this concentration range after exposure for similar periods of time [9,17,18,24]. Negative controls for this experiment included cells propagated in complete growth medium and vehicle-treated cells. Positive controls included cells exposed to 50% sodium lauryl sulphate (SDS) for 48 h since previous studies indicated that SDS induces a significant decrease in cell numbers and cell proliferation [41]. Subsequently, growth medium and PPV was discarded, and cells were fixed with 1% glutaraldehyde (100 μL) purchased from Merk (Darmstadt, Germany) before incubation for 15 min at room temperature. Glutaraldehyde was removed, and cells were stained using 0.1% crystal violet solution (100 μL) purchased from Merk (Darmstadt, Germany) and incubated at room temperature for 30 min. Afterwards, the crystal violet solution was discarded, and the 96-well plate was submersed under running water for 15 min [42]. The plate was then left to dry for 24 h and 0.2% Triton X-100 (200 μL) was added to solubilise the crystal violet stain at room temperature for 30 min [42]. The absorbance was then read at 570 nm using an EPOCH Microplate Reader (Biotek Instruments, Inc. (Winooski, Vermont, United States of America)) [42]. The data obtained were analysed using Microsoft Excel 2016 (Microsoft corporation, Washington, United States of America).

#### 4.2.2. Cell Morphology

##### Morphology Observation Using Light Microscopy

Light microscopy was used to evaluate and visualise the effects of PPV on MDA-MB-231, A549, and DU145 cells which were seeded into 24-well culture plates, at a cell density of 20,000 cells per well. The cells were incubated at 37 °C and 5% CO_2_ in a humified atmosphere for 24 h to allow for attachment. Subsequently, cells were exposed to PPV (10–100 μM) for 48 h or 72 h since previous research showed optimal activity in cancer cell lines [9,17,18,24]. The morphology of at least 100 cells was examined per condition in each experiment to quantify morphology. Aberrant morphological observations after exposure to PPV included shrunken cells, rounded cells, membrane blebbing, cells with lamellipodia-like protrusions, and cells revealing enlarged rounded morphology. An Axiovert 40 CFL microscope (Zeiss, Oberkochen, Germany) was used to capture images. Negative controls for this experiment included cells propagated in complete growth medium and vehicle-treated cells. Positive controls included cells exposed to 0.4 μM 2-Ethyl-17-hydroxy-13-methyl-7,8,9,11,12,13,14,15,16,17-decahydro-6-cyclopenta[a]phenanthren-3-yl sulphamate (ESE-ol) for 48 h since previous studies indicated that ESE-ol induces significant changes in cell morphology [43,44]. 

#### 4.2.3. Oxidative Stress 

##### Hydrogen Peroxide Production Using 2,7 Dichlorofluoresceindiacetate (DCFDA) (Fluorescent Microscopy) 

The effects of PPV on hydrogen peroxide (H_2_O_2_) production was used as an indicator of oxidative stress. A non-fluorescent probe, 2,7 dichlorofluoresceindiacetate (DCFDA), is oxidised by reactive oxygen species (ROS) to a fluorescent derivative, 2,7-dichlorofluorescein (DCF). Thus, DCFDA was used in this study as an indicator of oxidative stress and the effect of PPV on hydrogen peroxide production through detection of DCF using fluorescent microscopy with a maximum excitation and emission spectra of 495 nm and 529 nm, respectively [45]. 

MDA-MB-231, A549, and DU145 cells were seeded into 24-well culture plates at a density of 20,000 cells per well. The cells were incubated at 37 °C and 5% CO_2_ in a humified atmosphere for 24 h to allow for cell attachment. Subsequently, cells were exposed to PPV (10–150 μM) for 48 h or 72 h since previous research showed optimal activity in cancer cell lines [9,17,18,24]. Negative controls for this experiment included cells propagated in complete growth medium and vehicle-treated cells. Positive controls included cells exposed to 0.4 μM ESE-ol since previous studies have shown a significant increase in hydrogen peroxide production after exposure to ESE-ol [44]. Subsequently, cells were washed with phosphate buffer solution (PBS) before incubation with DCFDA (20 μM) for 25 min at 37 °C and 5% CO_2_ in a humified atmosphere. The wells were washed with PBS (0.5 mL) and PBS (500 μL) was added to each well. A Zeiss Axiovert CFL40 microscope, Zeiss Axiovert MRm monochrome camera (Zeiss, Oberkochen, Germany) and Zeiss filter 9 was operated to capture images of DCFDA-stained (green) cells. Fluorescence images were analysed using ImageJ software developed by the National Institutes of Health (Bethesda, Maryland, United States of America). The fluorescent intensity of at least 100 cells was evaluated per condition in each experiment using ImageJ software. 

### 4.3. Cell Cycle Progression and Cell Death Induction

#### Cell Cycle Analysis Using Propidium Iodide Staining (Flow Cytometry) 

The effects of PPV on cell cycle progression was evaluated using flow cytometry. Propidium iodide (PI) is used to stain DNA in order to quantify DNA correlated to each phase of the cell cycle (sub-G_1_, G_1_, S, G_2_M and endoreduplication) [46]. 

MDA-MB-231, A549, and DU145 cells were seeded into T25 cm^2^ culture flask at a density of 1,000,000 cells per flask. Thereafter, the flasks were incubated at 37 °C and 5% CO_2_ in a humified atmosphere for 24 h to allow for attachment. Subsequently, cells were exposed to PPV (10–150 μM) for 48 h or 72 h since previous research showed optimal activity in cancer cell lines [9,17,18,24]. Negative controls for this experiment included cells propagated in complete growth medium and vehicle-treated cells. Positive controls included cells exposed to 0.4 μM ESE-ol for 48 h since previous studies indicated that ESE-ol induces significant cell death as indicated by a sub-G_1_ peak [44]. Cells were then trypsinised and resuspended in 1 mL of complete growth medium [47]. Thereafter, samples were centrifugated for 5 min at 300*× g,* the supernatant was removed and the pellet of each sample was resuspended in 1 mL of ice-cold PBS containing 0.1% FCS [47]. Ice-cold ethanol (70%, 4 mL) was then added in a dropwise manner after which samples were stored at 4 °C for at least 24 h [47,48]. Samples were centrifuged for 5 min at 300*× g*; the supernatant was discarded and the pellet then resuspended in 1 mL PBS containing 40 μg/mL of PI, 100 μg/mL RNAse A and 0.1% triton X-100 [47]. Subsequently, samples were incubated at 37 °C and 5% CO_2_ in a humified atmosphere for 45 min. Propidium iodide fluorescence was measured with the cytoFLEX flow cytometer (Beckman Coulter, Inc. (Brea, California, United States of America)) available from the Institute for Cellular and Molecular Medicine (ICMM), University of Pretoria, South Africa. At least 10,000 events in each sample and data was analysed. Data from cell debris and aggregated cells was excluded from analyses [47]. Cell cycle distributions were calculated using FlowJo™ Software Version 10 (Becton, Dickinson and Company, 2019 (Ashland, Oregon, United States of America)) by assigning relative DNA content per cell to sub-G_1_, G_1_, S, and G_2_M phases [47]. As propidium iodide emits light at 617 nm, the data collected from the log forward detector number 3 were represented on the histograms derived on the *x*-axis [47]. 

### 4.4. Statistical Analysis

Three independent experiments were conducted for all techniques performed, where the mean and the standard deviation were calculated. Means are illustrated by using bar charts and standard deviations are shown with errors bars. A *p*-value < 0.05 calculated by means of the Student *t*-test was used for statistical significance and is indicated by an asterisk (*) using the Jamovi statistical software version 1.6 (The Jamovi project (2021) (Sydney, Australia). The fluorescent intensity of at least 100 cells was evaluated per condition using Image J software developed by the National Institutes of Health (Bethesda, Maryland, United States of America). Flow cytometry analysis involved at least 10,000 events in each sample and the data were analysed using FlowJo™ Software Version 10 (Becton, Dickinson, and Company, 2019 (Ashland, Oregon, United States of America)).

## 5. Conclusions

This study demonstrated that PPV exerts antiproliferative effects in a time- and dose-dependent manner in MDA-MB-231, A549, and DU145 cells. An increase in aberrant morphological changes including lamellipodia-like protrusions was observed in all three cell lines. H_2_O_2_ production increased in A549 cells at 48 h and in MDA-MB-231 and A549 at 72 h. Cell cycle analysis revealed that PPV exerted a cell line specific and time- and dose-dependent effect that increased the percentage of cells in the sub-G_1_ and endoreduplication peaks. Understanding these cell line specific effects will aid in the development of this compound and potential derivatives of this compound as an antiproliferative agent in cancer research. Future studies will involve further investigation into the molecular mechanism of PPV to clarify how PPV exerts these effects. 

## Figures and Tables

**Table 1:** The effects of PPV on cell cycle and cell death induction as a percentage of cells in each phase of the cell cycle on MDA-MB-231 cells at 48 h // 72 h. Statistical significance is represented by an * when using the Student *t*-test with a *p* value of 0.05 compared to cells propagated in growth medium.

48 h // 72 h					
	sub-G_1_	G_1_	S	G_2_/M	Endoreduplication
**Cells propagated in growth medium**	2.09 ± 0.61 // 1.65 ± 0.24	61.80 ± 2.4 2// 76.00 ± 0.80	6.90 ± 0.68 // 5.63 ± 0.48	15.77 ± 1.06 // 15.60 ± 0.70	0.41 ± 0.26 // 0.85 ± 0.13
**Vehicle-treated cells**	2.35 ± 0.49 // 2.19 ± 0.22	65.43 ± 2.22 // 70.77 ± 0.65 *	10.70 ± 0.60 * // 7.12 ± 1.87	19.40 ± 1.28 // 18.80 ± 0.46 *	0.48 ± 0.22 // 0.61 ± 0.22
**10 μM PPV-treated cells**	7.32 ± 0.72 *// 10.32 ± 0.57 *	59.10 ± 1.37 // 60.93 ± 1.01 *	7.32 ± 0.25 // 6.18 ± 0.65	17.53 ± 1.27 // 13.47 ± 0.99	1.50 ± 0.22 * // 4.28 ± 0.46 *
**50 μM PPV-treated cells**	9.00 ± 0.71 *// 7.12 ± 0.78 *	65.77 ± 1.53 *// 53.43 ± 1.32 *	10.16 ± 0.47 // 6.09 ± 0.68	13.60 ± 0.70 // 18.37 ± 1.00 *	1.47 ± 0.54 * // 10.83 ± 0.95 *
**100 μM PPV-treated cells**	7.36 ± 1.23 *// 11.25 ± 1.30 *	56.50 ± 0.85 // 54.07 ± 1.20 *	10.16 ± 0.47 * // 6.09 ± 0.68 *	21.80 ± 0.20 * // 16.37 ± 0.61	2.55 ± 1.27 * // 10.83 ± 0.95 *
**150 μM PPV-treated cells**	14.07 ± 0.65 *// 47.73 ± 1.46 *	60.33 ± 0.78 // 34.80 ± 1.57 *	6.71 ± 0.49 // 3.88 ± 0.32 *	16.03 ± 0.86 // 10.77 ± 0.21 *	1.47 ± 0.85 // 5.06 ± 0.53 *
**ESE-ol-treated cells**	28.90 ± 0.87 *// 25.87 ± 0.67 *	30.83 ± 1.07 * // 40.03 ± 0.72 *	10.47 ± 0.72 * // 8.39 ± 0.54 *	29.97 ± 0.81 * // 22.60 ± 1.39 *	0.18 ± 0.18 // 0.41 ± 0.08

**Table 2:** The effects of PPV on cell cycle and cell death induction as a percentage of cells in each phase of the cell cycle on A549 cells at 48 h // 72 h. Statistical significance is represented by an * when using the Student *t*-test with a *p* value of 0.05 compared to cells propagated in growth medium.

48 h // 72 h					
	sub-G_1_	G_1_	S	G_2_/M	Endoreduplication
**Cells propagated in growth medium**	2.93 ± 0.07 // 2.87 ± 0.74	71.63 ± 0.70 // 70.87 ± 1.01	6.78 ± 0.69 // 11.27 ± 1.26	18.30 ± 0.36 // 10.65 ± 2.09	0.89 ± 0.11 //0.08 ± 0.01
**Vehicle-treated cells**	3.42 ± 0.59 // 2.40 ± 0.02	70.73 ± 1.37 // 74.30 ± 1.68 *	9.04 ± 0.90 // 9.18 ± 0.72 *	17.87 ± 0.76 // 14.77 ± 1.29 *	0.59 ± 0.09 //0.08 ± 0.01
**10 μM PPV-treated cells**	7.53 ± 0.10 * // 4.24 ± 2.29	57.90 ± 0.82 * // 63.00 ± 1.39 *	5.97 ± 0.77 // 6.38 ± 0.37 *	25.17 ± 0.38 * // 16.63 ± 1.39 *	3.22 ± 0.31 * //3.03 ± 0.90 *
**50 μM PPV-treated cells**	11.23 ± 0.15 * // 8.45 ± 1.23 *	55.40 ± 0.66 *// 62.27 ± 1.62 *	7.78 ± 0.46 // 5.05 ± 0.61 *	23.30 ± 1.35 * // 12.03 ± 1.96	3.09 ± 0.66 * //8.37 ± 2.07 *
**100 μM PPV-treated cells**	13.33 ± 0.90 * // 5.12 ± 0.35 *	60.00 ± 0.53 * // 72.30 ± 2.50	7.78 ± 0.46 // 5.05 ± 0.61 *	16.43 ± 0.64 // 13.10 ± 1.65	2.58 ± 0.28 * //1.73 ± 0.40 *
**150 μM PPV-treated cells**	11.77 ± 0.32 * // 9.60 ± 0.53 *	68.77 ± 1.21 * // 66.23 ± 1.91 *	4.33 ± 0.42 * // 5.29 ± 0.78 *	14.23 ± 1.37 *// 11.80 ± 2.26 *	1.66 ± 0.74 //3.21 ± 0.97 *
**ESE-ol-treated cells**	35.97 ± 1.80 * // 24.50 ± 0.75 *	22.43 ± 0.21 * // 56.20 ± 1.31 *	7.72 ± 0.54 // 8.08 ± 2.33	29.50 ± 1.35 * // 13.87 ± 1.17	0.42 ± 0.42 //0.03 ± 0.02

**Table 3:** The effects of PPV on cell cycle and cell death induction as a percentage of cells in each phase of the cell cycle on DU145 cells at 48 h // 72 h. Statistical significance is represented by an * when using the Student t-test with a *p* value of 0.05 compared to cells propagated in growth medium.

48 h // 72 h					
	sub-G_1_	G_1_	S	G_2_/M	Endoreduplication
**Cells propagated in growth medium**	3.22 ± 0.91 // 1.41 ± 0.19	64.03 ± 0.96 // 76.70 ± 0.30	10.56 ± 0.82 // 5.47 ± 0.36	19.67 ± 0.78 // 16.67 ± 0.49	0.95 ± 0.03 // 0.52 ± 0.28
**Vehicle-treated cells**	3.16 ± 0.94 // 1.44 ± 0.07	65.90 ± 1.97 // 66.60 ± 2.10 *	11.30 ± 0.85 * // 9.80 ± 0.54 *	17.60 ± 0.89 // 18.90 ± 1.74	0.52 ± 0.36 // 0.75 ± 0.14
**10 μM PPV-treated cells**	12.83 ± 1.70 * // 14.30 ± 0.53 *	58.10 ± 0.26 * // 60.77 ± 2.31 *	7.47 ± 0.57 * // 9.34 ± 0.36 *	17.90 ± 0.17 * // 16.37 ± 0.93	2.15 ± 0.60 * // 3.63 ± 0.04 *
**50 μM PPV-treated cells**	10.91 ± 0.94 * // 10.90 ± 0.85 *	54.47 ± 0.81 * // 50.73 ± 1.26 *	7.15 ± 0.84 * // 7.35 ± 0.45 *	22.13 ± 1.43 // 20.27 ± 0.65 *	2.58 ± 0.35 * // 9.34 ± 0.51 *
**100 μM PPV-treated cells**	8.16 ± 0.27 * // 15.13 ± 0.32 *	63.10 ± 3.05 // 57.83 ± 0.70 *	7.15 ± 0.84 * // 7.35 ± 0.45 *	18.23 ± 1.31 * // 17.27 ± 0.78 *	1.76 ± 0.22 * // 4.57 ± 1.18 *
**150 μM PPV-treated cells**	8.87 ± 0.56 * // 23.97 ± 0.38 *	56.57 ± 0.55 * // 51.50 ± 0.87 *	9.62 ± 0.64 * // 5.21 ± 0.63	21.23 ± 0.91 // 15.67 ± 0.58	2.30 ± 0.82 // 4.62 ± 1.25 *
**ESE-ol-treated cells**	32.70 ± 1.51 * // 36.23 ± 0.81 *	27.67 ± 1.19 * // 38.03 ± 1.40 *	8.75 ± 0.62 * // 7.66 ± 0.33 *	28.53 ± 3.54 // 17.37 ± 0.76	0.52 ± 0.64 // 0.08 ± 0.05

## Data Availability

Data is contained within the article and Supplementary Material.

## Supplementary Materials

The following are available online at www.mdpi.com/1420-3049/26/21/6388, Supplementary 1; Figure S1: Spectrophotometry results of crystal violet staining demonstrating the effects of PPV (10–300 µM) on proliferation on MDA-MB-231 cells compared to A549- and DU145 cell lines at 24 h, Figure S2: Spectrophotometry results of crystal violet staining demonstrating the effects of PPV (10–300 µM) on proliferation on MDA-MB-231 cells compared to A549- and DU145 cell lines at 96 h. Supplementary 2; Figure S1: Light microscopy images of cell morphology demonstrating the effects of PPV ((10-150 µM) on cell morphology on MDA-MB-231 cells at 48 h at a magnification of ×10. Table S1: table displaying the effects of papaverine on morphology as percentage change when compared to cells propagated in growth medium on MDA-MB-231 at 48 h. Figure S2: Light microscopy images of cell morphology demonstrating the effects of PPV ((10–150 µM) on cell morphology on A549 cells at 48 h at a magnification of ×10. Table S2: table displaying the effects of papaverine on morphology as percentage change when compared to cells propagated in growth medium on A549 at 48 h. Figure S3: Light microscopy images of cell morphology demonstrating the effects of PPV ((10–150 µM) on cell morphology on DU145 cells at 48 h at a magnification of ×10 Table S3: table displaying the effects of papaverine on morphology as percentage change when compared to cells propagated in growth medium on DU145 at 48 h. Figure S4: Light microscopy images of cell morphology demonstrating the effects of PPV ((10–150 µM) on cell morphology on MDA-MB-231 cells at 72 h at a magnification of ×10 Table S4: table displaying the effects of papaverine on morphology as percentage change when compared to cells propagated in growth medium on MDA-MB-231 at 72 h. Figure S5: Light microscopy images of cell morphology demonstrating the effects of PPV ((10–150 µM) on cell morphology on A549 cells at 72 h at a magnification of ×10 Table S5: table displaying the effects of papaverine on morphology as percentage change when compared to cells propagated in growth medium on A549 at 72 h. Figure S6: Light microscopy images of cell morphology demonstrating the effects of PPV ((10–150 µM) on cell morphology on DU145 cells at 72 h at a magnification of ×10 Table S6: table displaying the effects of papaverine on morphology as percentage change when compared to cells propagated in growth medium on DU145 at 72 h. Figure S7. Light microscopy results demonstrating the effects of ESE-ol used as a positive control on cell morphology. Supplementary 3; Table S1. table displaying the effects of papaverine on oxidative stress as a change of fluorescence intensity relative to the fluorescence intensity of cells propagated in growth medium on MDA-MB-231-, A549- and DU145 cell lines at 48 h. Figure S1. Fluorescence staining showing H_2_O_2_ production in MDA-MB-231 cells after 48 h. Figure S2. Fluorescence staining showing H_2_O_2_ production in A549 cells after 48 h. Figure S3. Fluorescence staining showing H_2_O_2_ production in MDA-MB-231 cells after 48 h. Table S2. table displaying the effects of papaverine on oxidative stress as a change of fluorescence intensity relative to the fluorescence intensity of cells propagated in growth medium on MDA-MB-231-, A549- and DU145 cell lines at 72 h. Figure S4. Fluorescence staining showing H_2_O_2_ production in MDA-MB-231 cells after 72 h. Figure S5. Fluorescence staining showing H_2_O_2_ production in A549 cells after 72 h. Figure S6. Fluorescence staining showing H_2_O_2_ production in MDA-MB-231 cells after 72 h. Supplementary 4; Figure S1. Flow cytometry results demonstrating the effects of PPV (10–150 µM) on the cell cycle on MDA-MB-231-, A549- and DU145 cells at 48 h. Figure S2. Cell cycle progression of MDA-MB-231 cells treated with PPV (10–150 µM) at 48 h. Figure S3. Cell cycle progression of A549 cells treated with PPV (10–150 µM) at 48 h. Figure S4. Cell cycle progression of DU145 cells treated with PPV (10–150 µM) at 48 h. Figure S5. Flow cytometry results demonstrating the effects of PPV (10–150 µM) on the cell cycle on MDA-MB-231-, A549- and DU145 cells at 72 h. Figure S6. Cell cycle progression of MDA-MB-231 cells treated with PPV (10–150 µM) at 72 h. Figure S7. Cell cycle progression of A549 cells treated with PPV (10–150 µM) at 72 h. Figure S8. Cell cycle progression of DU145 cells treated with PPV (10–150 µM) at 72 h.

## Sample Availability

Samples of the compounds are not available from the authors.
