# Supplementary material for: In Vitro Effects of Papaverine on Cell Proliferation, Reactive Oxygen Species, and Cell Cycle Progression in Cancer Cells
Source: Molecules. 2021 Oct 22;26(21):6388. doi: 10.3390/molecules26216388 (PMC8587410; doi:10.3390/molecules26216388)
Supplement: Supplementary file 1 [file molecules-26-06388-s001.zip › molecules-1368532-supplementary/molecules-1368532-supplementary/Supplementary data/Supplementary 1.pdf]

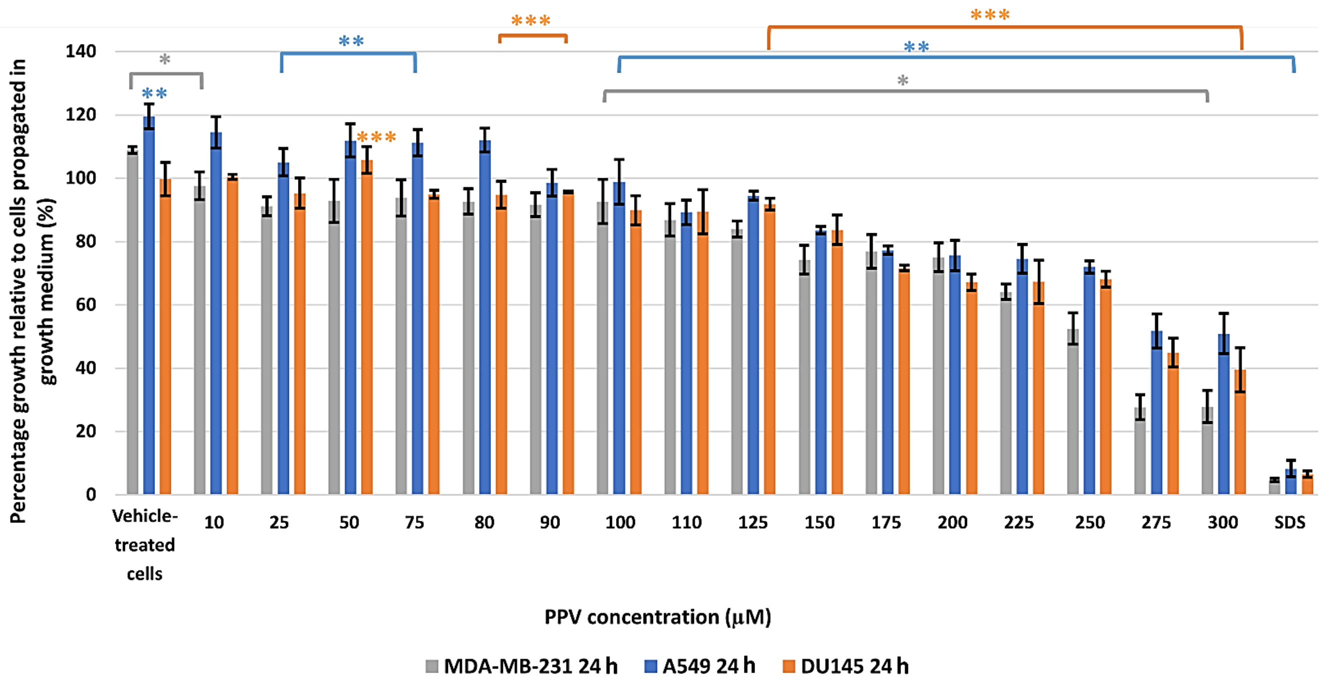

Figure S1. Spectrophotometry results of crystal violet staining demonstrating the effects of PPV (10-300 µM) on proliferation on MDA-MB-231 cells compared to A549- and DU145 cell lines at 24 h.The average of 3 independent experiments is represented by the graph with error bars indicating standard deviation. The statistical significance of MDA-MB-231 cells is indicated with an asterisk (*), the statistical significance of A549 cells is indicated with two asterisks (**) and the statistical significance of DU145 cells is indicated with three asterisks (***). Statistical significance is represented by an * when using the student t-test with a P value of 0.05 compared to cells propagated in growth medium.

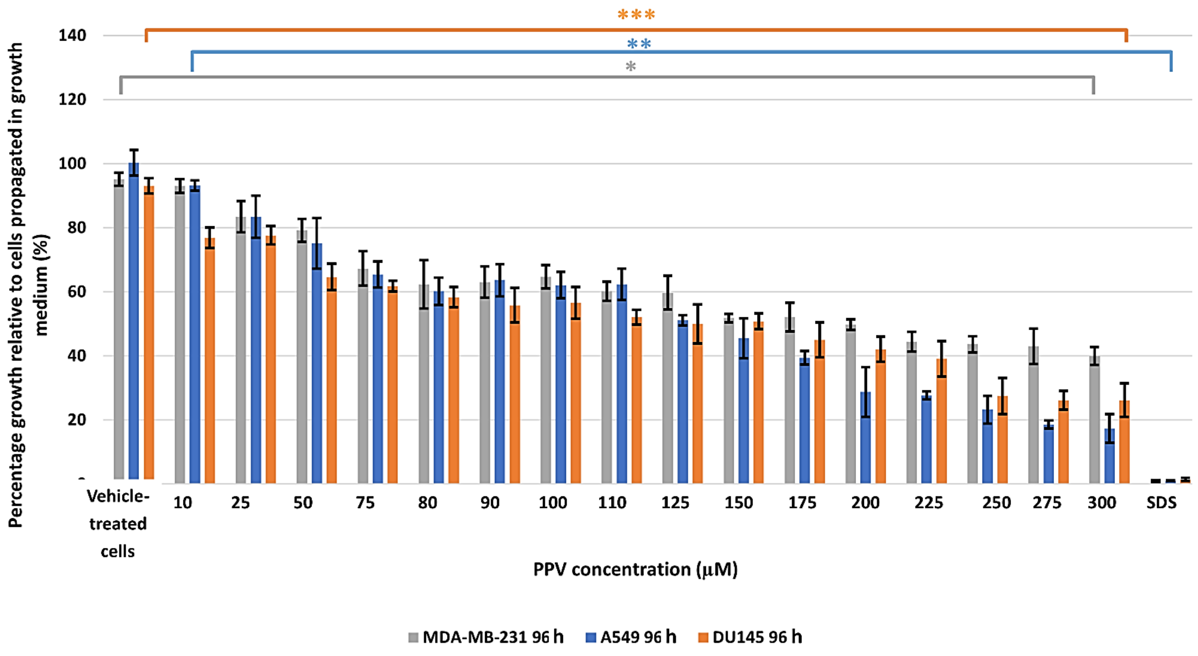

Figure S2. Spectrophotometry results of crystal violet staining demonstrating the effects of PPV (10-300 µM) on proliferation on MDA-MB-231 cells compared to A549- and DU145 cell lines at 96 h. The average of 3 independent experiments is represented by the graph with error bars indicating standard deviation. The

statistical significance of MDA-MB-231 cells is indicated with an asterisk (*), the statistical significance of A549 cells is indicated with two asterisks (**) and the statistical significance of DU145 cells is indicated with three asterisks (***). Statistical significance is represented by an * when using the student t-test with a P value of 0.05 compared to cells propagated in growth medium.
