# Supplementary material for: In Vitro Effects of Papaverine on Cell Proliferation, Reactive Oxygen Species, and Cell Cycle Progression in Cancer Cells
Source: Molecules. 2021 Oct 22;26(21):6388. doi: 10.3390/molecules26216388 (PMC8587410; doi:10.3390/molecules26216388)
Supplement: Supplementary file 1 [file molecules-26-06388-s001.zip › molecules-1368532-supplementary/molecules-1368532-supplementary/Supplementary data/Supplementary 2.pdf]

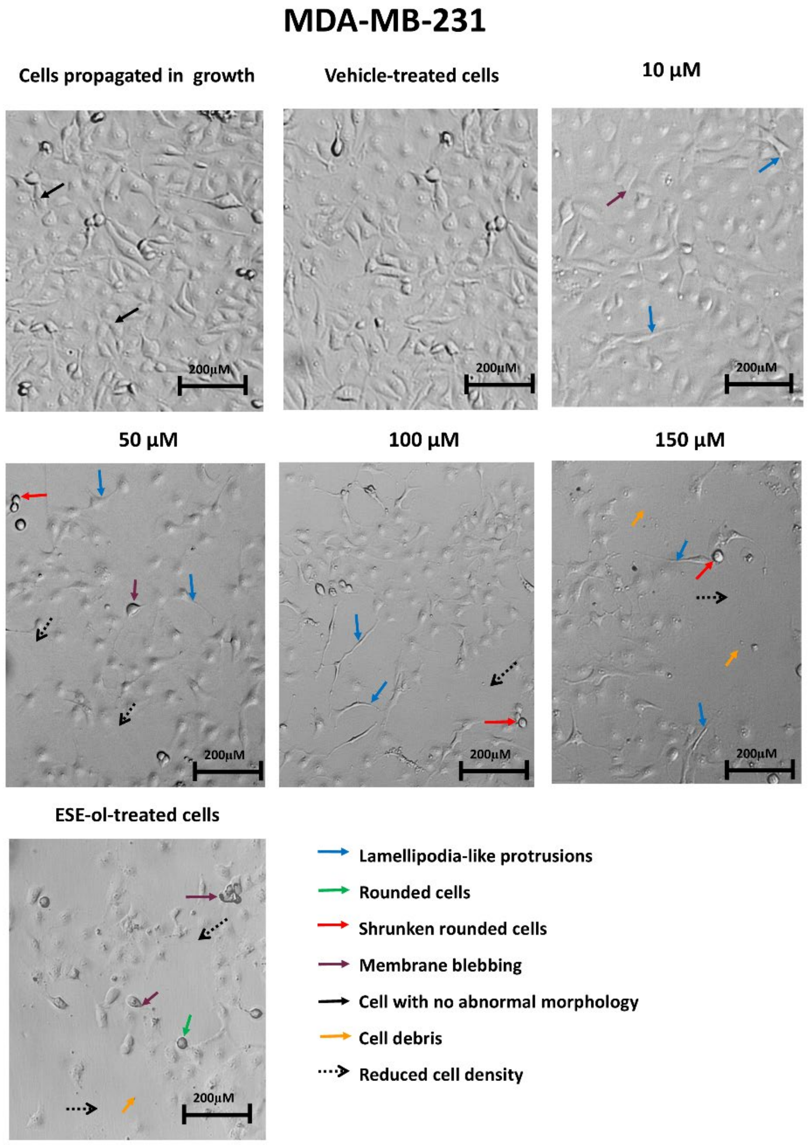

Figure S1. Light microscopy images of cell morphology demonstrating the effects of PPV ((10-150 μM) on cell morphology on MDA-MB-231 cells at 48 h at a magnification of x10. Blue arrows indicate the lamellipodia-like protrusions, green arrows indicate rounded cells, red arrows indicate shrunken rounded cells, purple arrows indicate cells exhibiting membrane blebbing, black solid arrows indicate cells with no abnormal morphology, yellow arrows indicate cell debris and black dashed arrows indicate areas exhibiting reduced cell density.

Table S1: table displaying the effects of papaverine on morphology as percentage change when compared to cells propagated in growth medium on MDA-MB-231 at 48 h. Statistical significance is represented by an * when using the student *t*-test with a *P* value of 0.05 compared to cells propagated in growth medium.

	**Cells propagate d in growth medium**	**Vehicle-treated cells**	**10 μM PPV-treated cells**	**50 μM PPV-treated cells**	**100 μM PPV-treated cells**	**150 μM PPV-treated cells**	**ESE-ol-treated cells**
**Cells demonstrating**	49.75 ± 0.96	55.00 ± 0.82	38.33 ± 0.58	40.00 ± 1.00*	32.00 ± 1.00*	32.33 ± 2.08*	32.00 ± 1.00*

**no abnormal morphology**							
**Shrunken cells demonstrating rounded morphology**	10.00 ± 1.15	9.75 ± 1.71	21.00 ± 1.73*	16.33 ± 2.52	21.33 ± 1.53*	22.33 ± 0.58*	21.67 ± 0.58*
**Cells demonstrating rounded morphology**	34.75 ± 0.96	31.50 ± 1.73	23.33 ± 1.15*	19.33 ± 1.15*	17.67 ± 2.52	15.33 ± 1.53	15.33 ± 0.58*
**Cells demonstrating membrane blebbing**	1.00 ± 0.82	0.00 ± 0.00	1.67 ± 0.58	1.67 ± 0.58	2.00 ± 1.00	2.33 ± 0.58*	3.33 ± 1.53
**Cells demonstrating lamellipodia-like protrusions**	6.25 ± 0.96	5.75 ± 0.50	18.67 ± 1.15*	25.67 ± 0.58*	28.67 ± 0.58*	30.33 ± 0.58*	30.00 ± 1.00*
**Cells demonstrating enlarged rounded morphology**	0.00 ± 0.00	0.00 ± 0.00	0.00 ± 0.00	0.00 ± 0.00	0.00 ± 0.00	0.00 ± 0.00	0.00 ± 0.00
**Cells demonstrating enlarged morphology**	0.00 ± 0.00	0.00 ± 0.00	0.00 ± 0.00	0.00 ± 0.00	0.00 ± 0.00	0.00 ± 0.00	0.00 ± 0.00

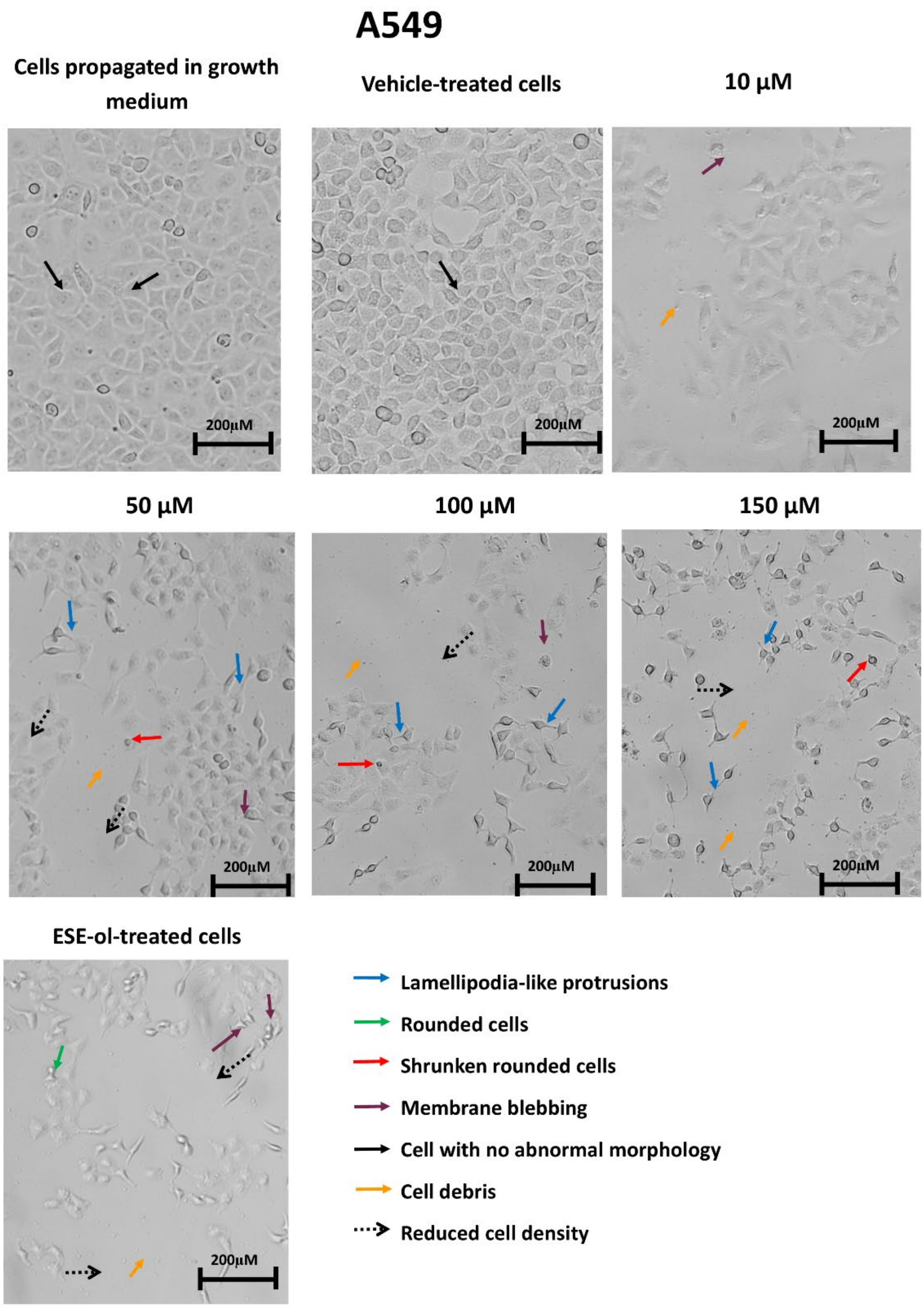

Figure 2. Light microscopy images of cell morphology demonstrating the effects of PPV ((10-150 µM) on cell morphology on A549 cells at 48 h at a magnification of x10. Blue arrows indicate the lamellipodia-like protrusions, green arrows indicate rounded cells, red arrows indicate shrunken rounded cells, purple arrows indicate cells exhibiting membrane blebbing, black solid arrows indicate cells with no abnormal morphology, yellow arrows indicate cell debris and black dashed arrows indicate areas exhibiting reduced cell density.

Table 2: table displaying the effects of papaverine on morphology as percentage change when compared to cells propagated in growth medium on A549 cells at 48 h. Statistical significance is represented by an * when using the student *t*-test with a *P* value of 0.05 compared to cells propagated in growth medium.

	**Cells propagat ed in**	**Vehicle-treated cells**	**10 µM PPV-treated cells**	**50 µM PPV-treated cells**	**100 µM PPV-treated cells**	**150 µM PPV-treated cells**	**ESE-ol-treated cells**

	growth medium						
**Cells demonstrating no abnormal morphology**	59.67 ± 0.58	60.33 ± 0.58	53.33 ± 1.15*	42.00 ± 1.73*	39.67 ± 0.58*	39.00 ± 1.00*	19.00 ± 0.58*
**Shrunken cells demonstrating rounded morphology**	0.00 ± 0.00	0.00 ± 0.00	17.67 ± 0.58*	18.00 ± 1.00*	13.00 ± 1.00*	13.00 ± 1.00*	37.33 ± 0.58*
**Cells demonstrating rounded morphology**	31.67 ± 0.58	33.33 ± 0.58*	3.33 ± 1.53*	1.67 ± 0.58	2.67 ± 0.58	3.33 ± 0.58*	12.67 ± 0.58*
**Cells demonstrating membrane blebbing**	1.33 ± 0.58	0.33 ± 0.58	2.67 ± 0.58*	3.00 ± 0.00*	3.00 ± 1.00	2.00 ± 0.00	5.67 ± 0.58*
**Cells demonstrating lamellipodia-like protrusions**	7.00 ± 1.00	5.00 ± 2.00	18.00 ± 1.00*	32.33 ± 0.58*	36.67 ± 0.58*	39.00 ± 1.00*	19.67 ± 0.58*
**Cells demonstrating enlarged rounded morphology**	0.33 ± 0.58	1.00 ± 1.00	5.00 ± 1.00*	3.00 ± 0.00*	3.67 ± 0.58*	2.00 ± 2.00*	3.33 ± 1.53
**Cells demonstrating enlarged morphology**	0.00 ± 0.00	0.00 ± 0.00	0.00 ± 0.00	0.00 ± 0.00	1.33 ± 0.58*	0.67 ± 0.58	2.33 ± 0.58*

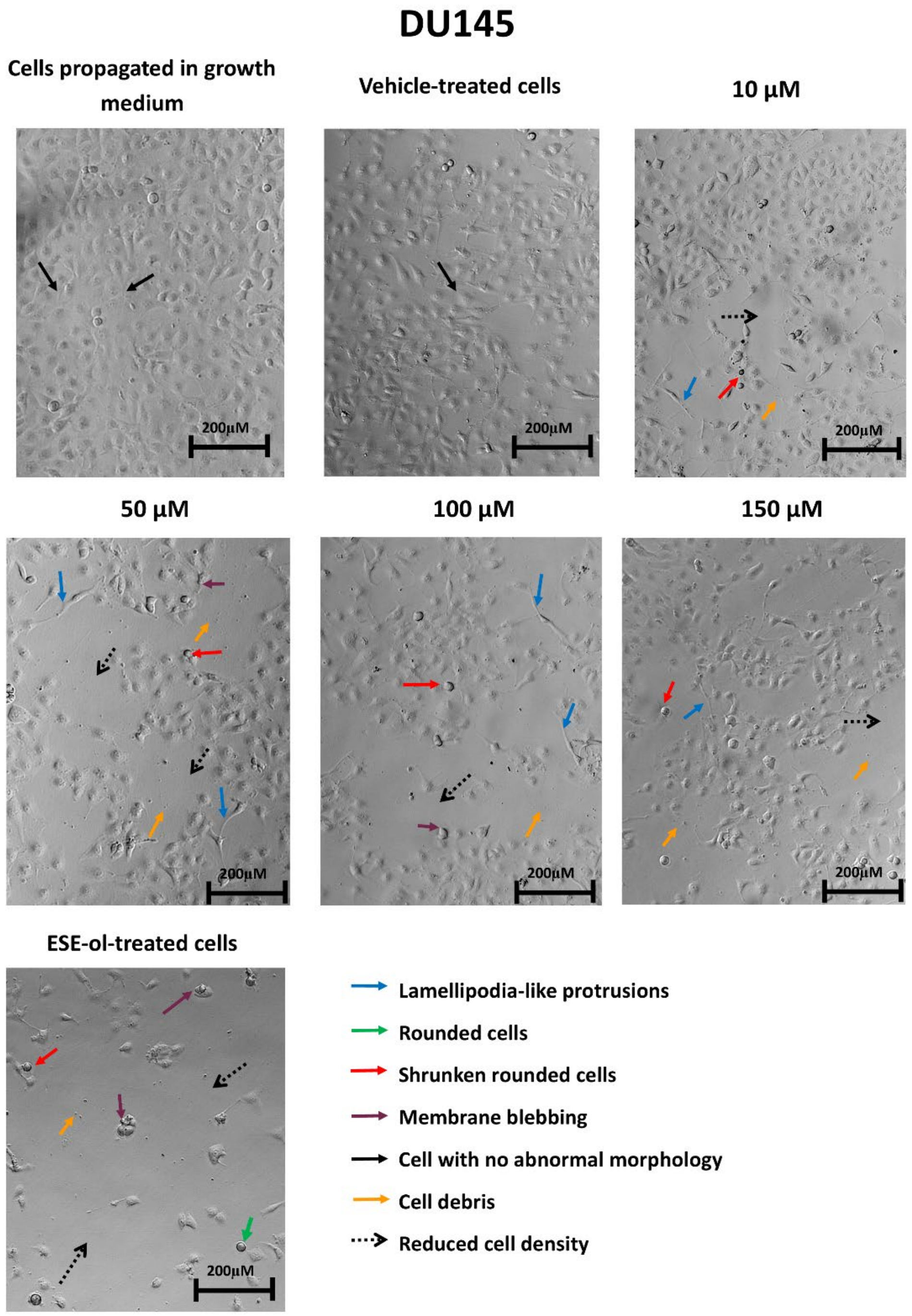

Table 3: table displaying the effects of papaverine on morphology as percentage change when compared to cells propagated in growth medium on DU145 cells at 48 h. Statistical significance is represented by an * when using the student *t*-test with a *P* value of 0.05 compared to cells propagated in growth medium.

	Cells propagated in growth medium	Vehicle-treated cells	10 µM PPV-treated cells	50 µM PPV-treated cells	100 µM PPV-treated cells	150 µM PPV-treated cells	ESE-ol-treated cells
**Cells demonstrating no abnormal morphology**	54.67 ± 1.53	56.00 ± 1.00	40.33 ± 0.58*	32.00 ± 1.73*	33.33 ± 1.15*	34.67 ± 2.31*	28.33 ± 1.15*
**Shrunken cells demonstrating rounded morphology**	6.00 ± 1.00	5.33 ± 0.58	11.00 ± 1.00*	24.00 ± 1.73*	24.00 ± 1.73*	17.33 ± 0.58*	32.00 ± 2.00*
**Cells demonstrating rounded morphology**	27.00 ± 2.00	30.00 ± 1.00	6.67 ± 0.58*	7.33 ± 0.58*	4.67 ± 1.15*	6.33 ± 0.58*	4.00 ± 1.00*
**Cells demonstrating membrane blebbing**	0.00 ± 0.00	0.33 ± 0.58	4.33 ± 1.15*	3.33 ± 1.15	2.67 ± 0.58*	3.67 ± 0.58	7.67 ± 1.15*
**Cells demonstrating lamellipodia-like protrusions**	9.00 ± 1.00	6.67 ± 0.58	35.67 ± 1.52*	31.00 ± 1.00*	34.33 ± 2.08*	35.67 ± 1.53*	24.00 ± 1.73*
**Cells demonstrating enlarged rounded morphology**	2.00 ± 1.00	1.33 ± 0.58	1.33 ± 1.15	2.00 ± 1.00	1.00 ± 1.00	1.33 ± 0.58	3.33 ± 1.15
**Cells demonstrating enlarged morphology**	1.33 ± 1.15	0.33 ± 0.58	0.67 ± 1.15	0.33 ± 0.58	0.00 ± 0.00	1.00 ± 1.00	0.67 ± 1.15

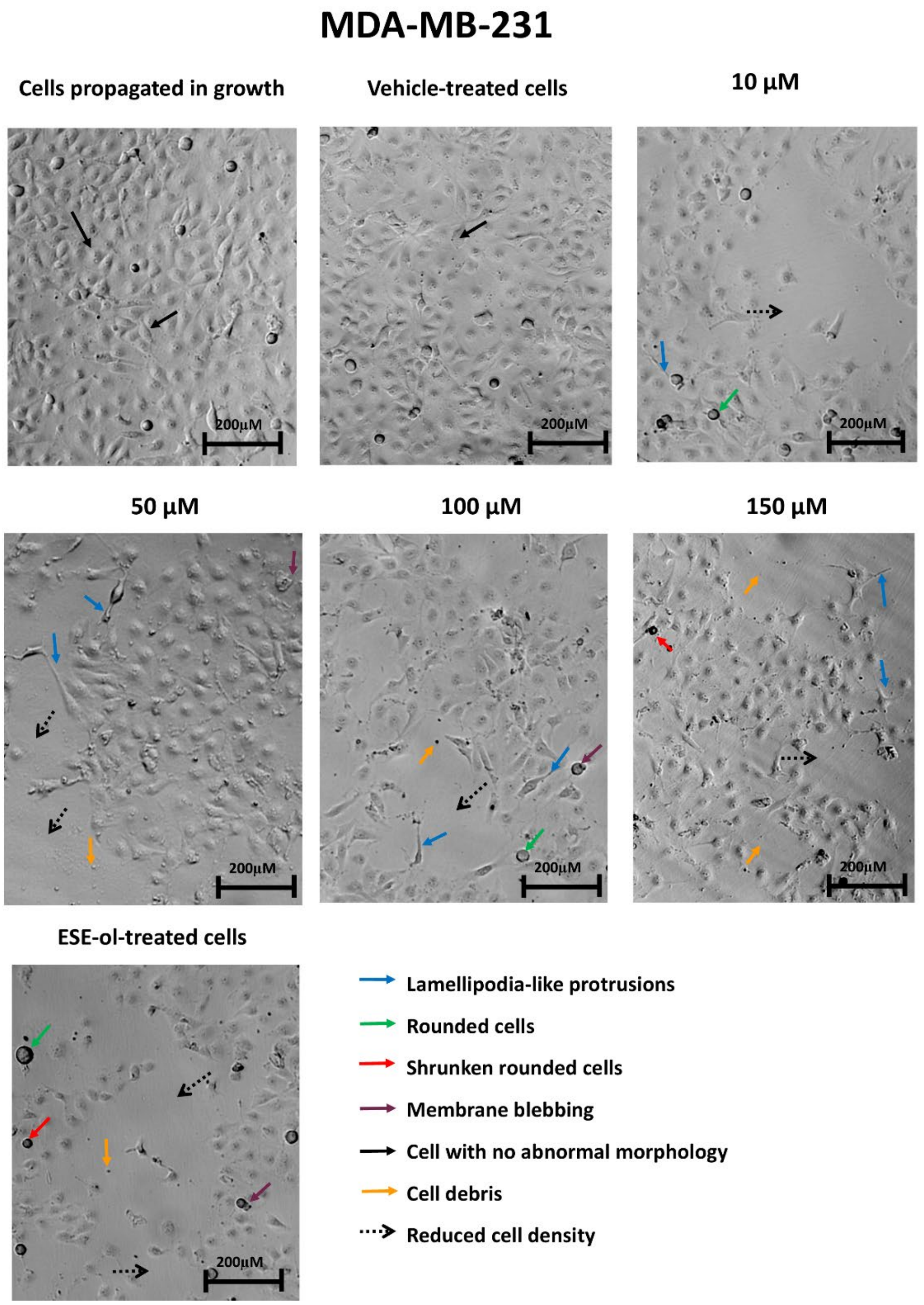

Figure 4. Light microscopy images of cell morphology demonstrating the effects of PPV ((10-150 µM) on cell morphology on MDA-MB-231 cells at 72 h at a magnification of x10. Blue arrows indicate the lamellipodia-like protrusions, green arrows indicate rounded cells, red arrows indicate shrunken rounded cells, purple arrows indicate cells exhibiting membrane blebbing, black solid arrows indicate cells with no abnormal morphology, yellow arrows indicate cell debris and black dashed arrows indicate areas exhibiting reduced cell density

Table 4: table displaying the effects of papaverine on morphology as percentage change when compared to cells propagated in growth medium on MDA-MB-231 cells at 72 h. Statistical significance is represented by an * when using the student *t*-test with a *P* value of 0.05 compared to cells propagated in growth medium.

	Cells propagated in growth medium	Vehicle-treated cells	10 µM PPV-treated cells	50 µM PPV-treated cells	100 µM PPV-treated cells	150 µM PPV-treated cells	ESE-ol-treated cells
**Cells demonstrating no abnormal morphology**	52.33 ± 1.15	55.33 ± 0.58*	40.33 ± 0.58*	36.67 ± 0.58*	35.00 ± 1.00*	34.00 ± 1.00*	27.67 ± 1.15*
**Shrunken cells demonstrating rounded morphology**	7.00 ± 1.00	5.67 ± 0.58	13.33 ± 0.58*	15.33 ± 1.15*	17.67 ± 0.58*	19.67 ± 0.58*	18.67 ± 1.53*
**Cells demonstrating rounded morphology**	26.33 ± 1.15	25.00 ± 1.00	10.67 ± 0.58*	10.33 ± 0.58*	6.67 ± 0.58*	5.67 ± 0.58*	16.67 ± 0.58*
**Cells demonstrating membrane blebbing**	1.33 ± 1.15	1.33 ± 0.58	2.33 ± 0.58	2.67 ± 1.15	2.00 ± 1.00	2.67 ± 1.15	4.33 ± 0.58
**Cells demonstrating lamellipodia-like protrusions**	7.00 ± 1.00	9.00 ± 1.00	31.00 ± 1.00	33.00 ± 1.00*	35.67 ± 0.58*	34.00 ± 1.00*	27.00 ± 1.00*
**Cells demonstrating enlarged rounded morphology**	3.67 ± 1.15	2.33 ± 0.58*	1.00 ± 1.00	0.67 ± 0.58	1.00 ± 1.00	1.67 ± 1.15	2.33 ± 0.58*
**Cells demonstrating enlarged morphology**	2.33 ± 0.58	1.33 ± 0.58	1.33 ± 0.58	1.33 ± 0.58	2.00 ± 1.00	2.33 ± 0.58	3.33 ± 0.58

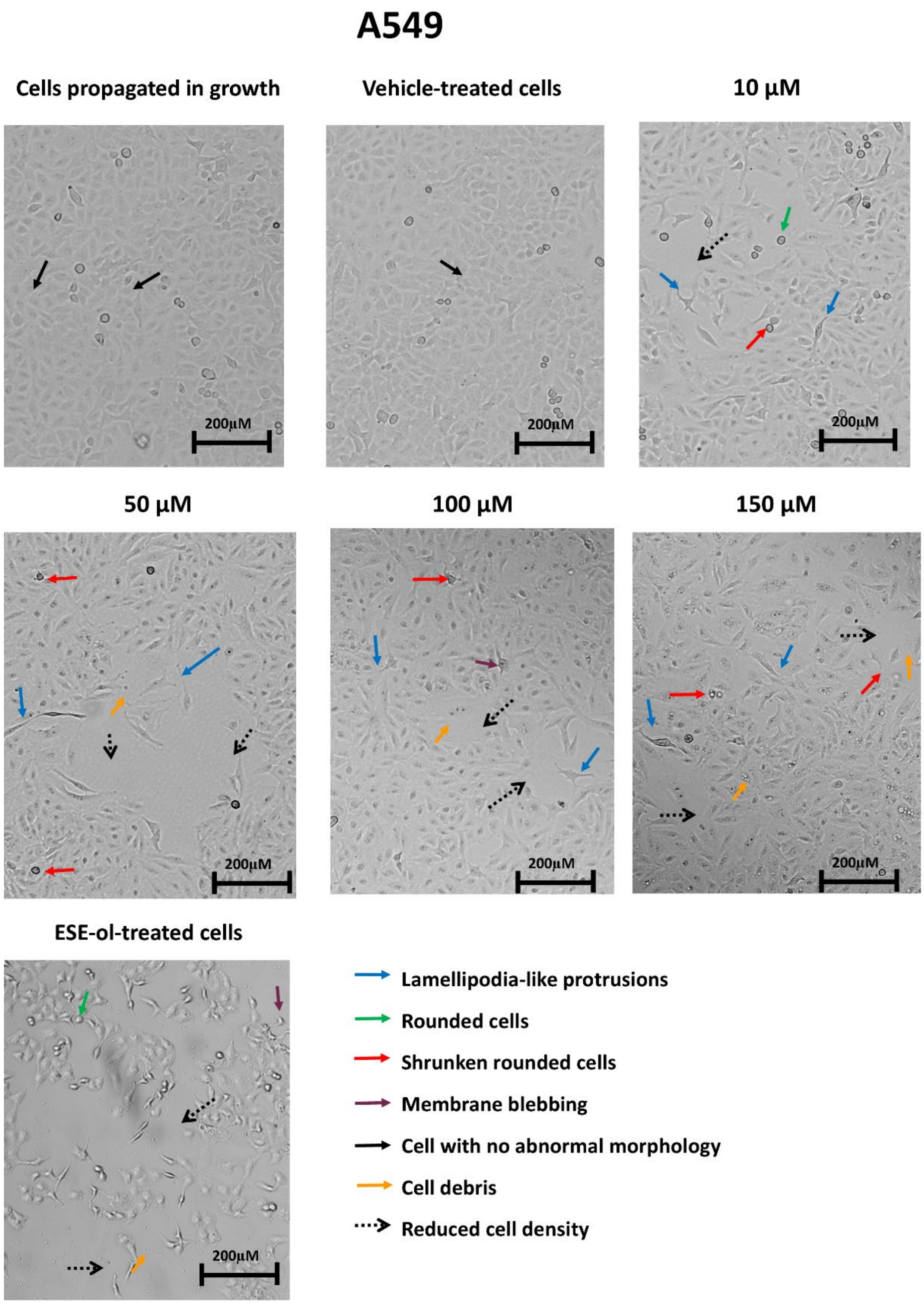

Figure 5. Light microscopy images of cell morphology demonstrating the effects of PPV ((10-150 µM) on cell morphology on A549 cells at 72 h at a magnification of x10. Blue arrows indicate the lamellipodia-like protrusions, green arrows indicate rounded cells, red arrows indicate shrunken rounded cells, purple arrows indicate cells exhibiting membrane blebbing, black solid arrows indicate cells with no abnormal morphology, yellow arrows indicate cell debris and black dashed arrows indicate areas exhibiting reduced cell density.

Table 5: table displaying the effects of papaverine on morphology as percentage change when compared to cells propagated in growth medium on A549 cells at 72 h. Statistical significance is represented by an * when using the student *t*-test with a *P* value of 0.05 compared to cells propagated in growth medium.

	Cells propagated in growth medium	Vehicle-treated cells	10 µM PPV-treated cells	50 µM PPV-treated cells	100 µM PPV-treated cells	150 µM PPV-treated cells	ESE-ol-treated cells
**Cells demonstrating no abnormal morphology**	60.00 ± 1.00	58.33 ± 1.53	50.33 ± 0.58*	45.00 ± 1.00*	40.33 ± 0.58*	39.00 ± 1.00*	18.33 ± 0.58*
**Shrunken cells demonstrating rounded morphology**	3.33 ± 0.58	4.67 ± 1.53	11.00 ± 1.00*	15.00 ± 1.00*	19.00 ± 1.00*	19.33 ± 1.15*	30.33 ± 1.53*
**Cells demonstrating rounded morphology**	28.67 ± 1.15	29.33 ± 0.58	23.00 ± 1.00*	19.33 ± 0.58*	15.67 ± 0.58*	13.67 ± 1.53*	10.33 ± 0.58*
**Cells demonstrating membrane blebbing**	0.00 ± 0.00	0.00 ± 0.00	2.00 ± 1.00*	3.33 ± 1.53*	3.33 ± 0.58*	3.33 ± 1.53*	11.00 ± 1.00*
**Cells demonstrating lamellipodia-like protrusions**	6.00 ± 1.00	5.67 ± 0.58	12.00 ± 1.00*	15.67 ± 2.08*	19.33 ± 0.58*	23.00 ± 0.00*	27.33 ± 2.08*
**Cells demonstrating enlarged rounded morphology**	2.00 ± 1.00	2.00 ± 0.00	1.67 ± 0.58	1.00 ± 0.00	1.67 ± 0.58	1.67 ± 0.58	2.33 ± 0.58
**Cells demonstrating enlarged morphology**	0.00 ± 0.00	0.00 ± 0.00	0.00 ± 0.00	0.67 ± 0.58	0.67 ± 0.58	0.00 ± 0.00	0.33 ± 0.58

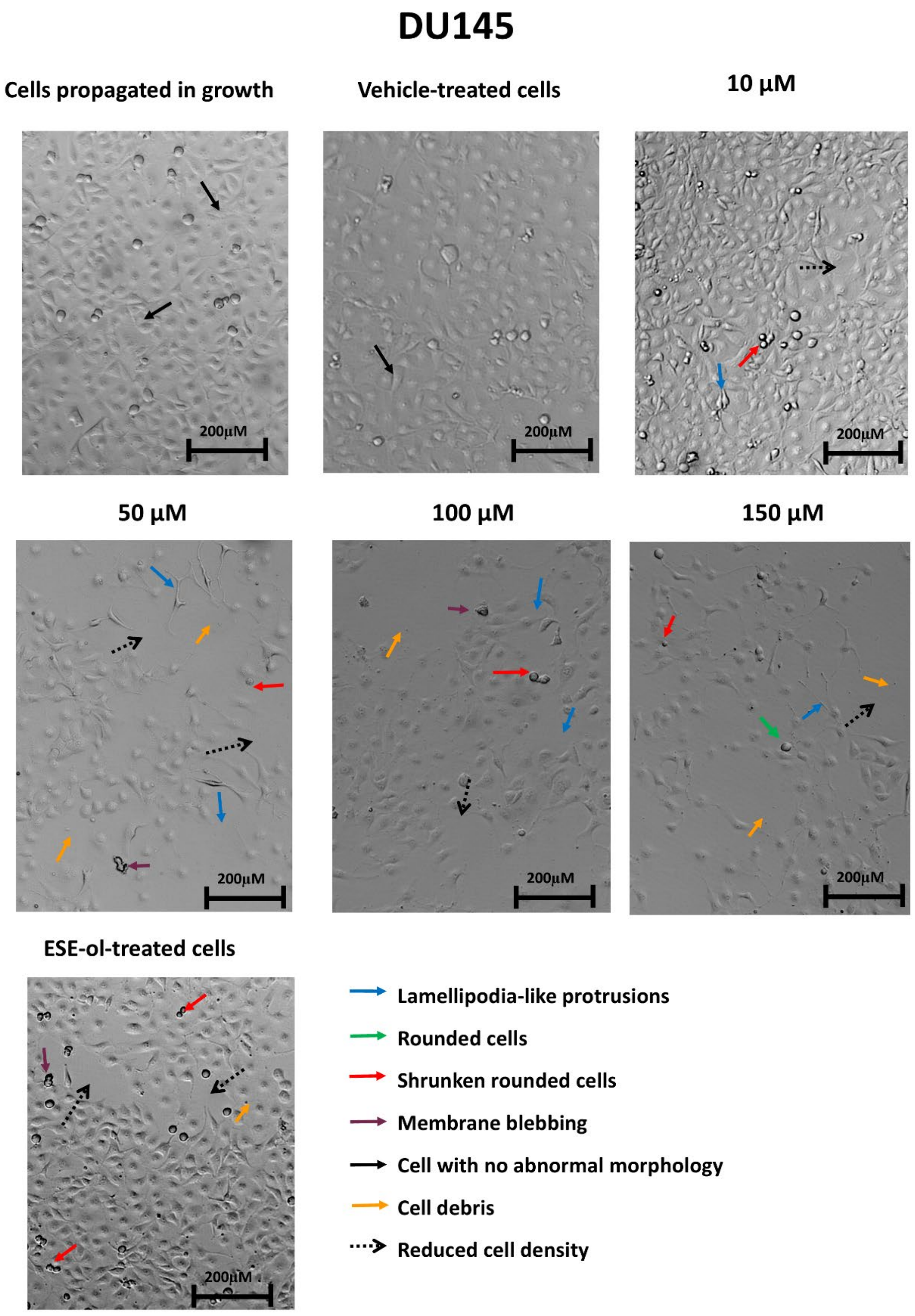

Figure 6. Light microscopy images of cell morphology demonstrating the effects of PPV ((10-150 µM) on cell morphology on DU148 cells at 72 h at a magnification of x10. Blue arrows indicate the lamellipodia-like protrusions, green arrows indicate rounded cells, red arrows indicate shrunken rounded cells, purple arrows indicate cells exhibiting membrane blebbing, black solid arrows indicate cells with no abnormal morphology, yellow arrows indicate cell debris and black dashed arrows indicate areas exhibiting reduced cell density.

Table 6: table displaying the effects of papaverine on morphology as percentage change when compared to cells propagated in growth medium on DU145 cells at 72 h. The statistical significance is represented by an * when data showed a 95% confidence level when compared to cells propagated in growth medium.

	Cells propagated in growth medium	Vehicle-treated cells	10 µM PPV-treated cells	50 µM PPV-treated cells	100 µM PPV-treated cells	150 µM PPV-treated cells	ESE-ol-treated cells
**Cells demonstrating no abnormal morphology**	57.67 ± 0.58	52.67 ± 1.53*	50.67 ± 0.58	47.33 ± 0.58*	47.33 ± 1.15*	45.67 ± 2.08	31.00 ± 1.00*
**Shrunken cells demonstrating rounded morphology**	5.33 ± 0.58	6.33 ± 0.58	7.33 ± 0.58	8.00 ± 1.00*	12.00 ± 1.00*	13.00 ± 1.00*	18.33 ± 1.53*
**Cells demonstrating rounded morphology**	23.33 ± 1.53	26.00 ± 1.00*	19.00 ± 1.00*	7.33 ± 0.58*	6.67 ± 0.58*	5.67 ± 0.58*	6.33 ± 0.58*
**Cells demonstrating membrane blebbing**	1.33 ± 1.15	0.67 ± 0.58	3.00 ± 1.00*	1.67 ± 0.58	2.00 ± 1.00	2.00 ± 1.00	10.00 ± 1.00*
**Cells demonstrating lamellipodia-like protrusions**	11.00 ± 1.00	11.67 ± 1.53	17.00 ± 1.00*	33.00 ± 1.00*	32.00 ± 1.00*	31.00 ± 1.00*	30.00 ± 1.00*
**Cells demonstrating enlarged rounded morphology**	1.33 ± 0.58	2.33 ± 0.58	2.00 ± 0.00	2.00 ± 0.00	0.00 ± 0.00	2.00 ± 1.00	3.67 ± 1.53
**Cells demonstrating enlarged morphology**	0.00 ± 0.00	0.33 ± 0.58	1.00 ± 1.00	0.67 ± 0.58	0.00 ± 0.00	0.67 ± 0.58	0.67 ± 1.15

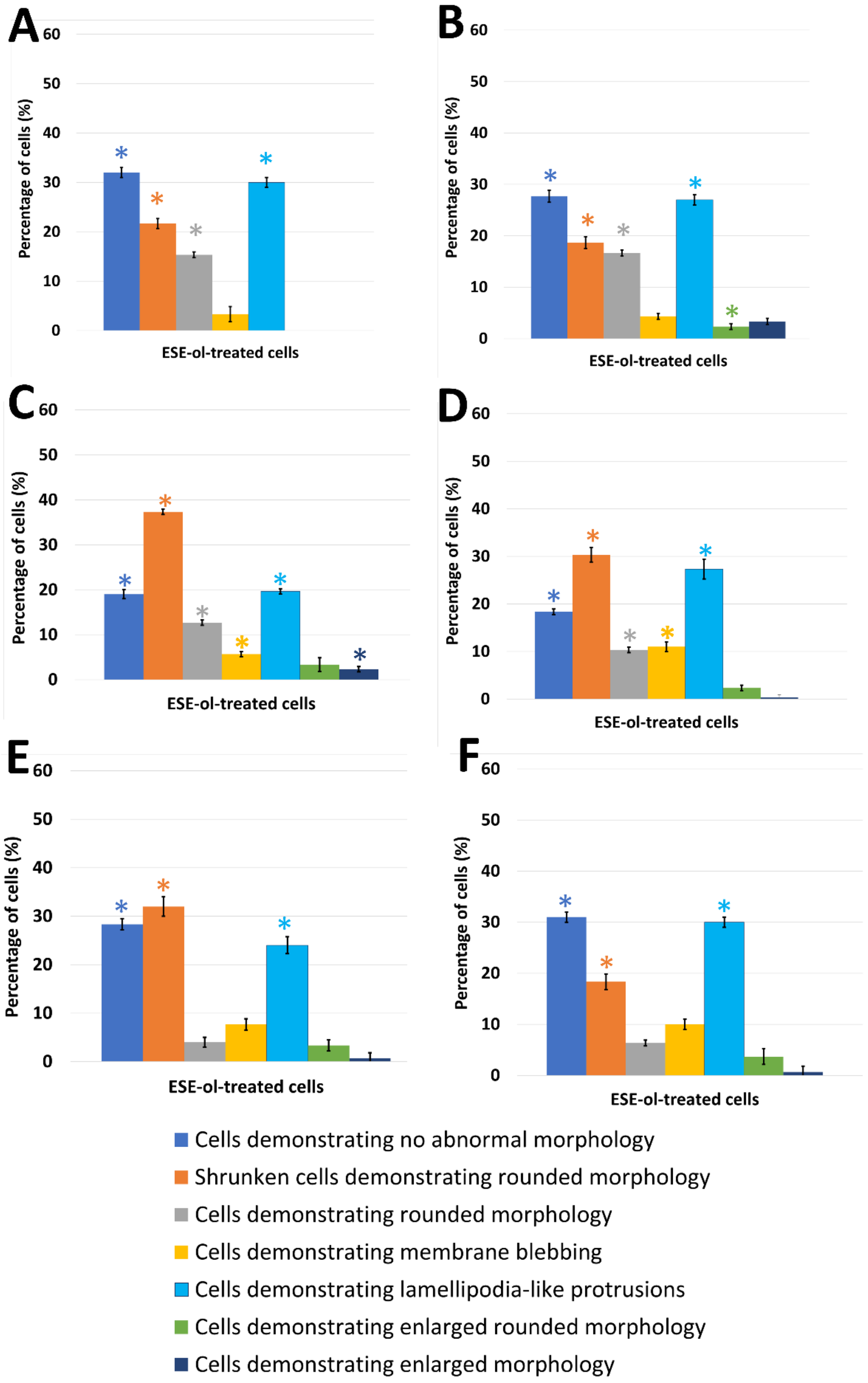

Figure 7. Light microscopy results demonstrating the effects of ESE-ol used as a positive control on cell morphology on MDA-MB-231 cells at 48 h (A)and 72 h (B), A549 cells at 48 h (C)and 72 h (D) and DU 145 cells at 48 h (E)and 72 h (F). The blue bar represents the percentage of cells demonstrating no abnormal morphology, the orange bar represents the percentage of shrunken cells demonstrating rounded morphology, the grey bar represents the percentage of cells demonstrating rounded morphology; the yellow bar represents the percentage of cells demonstrating membrane blebbing; the light blue bar represents the percentage of cells demonstrating lamellipodia-like protrusions; the green bar represents the percentage of cells demonstrating enlarged rounded morphology, and the dark blue bar represents the percentage of cells demonstrating enlarged morphology. Statistical significance is represented by an * when using the student t-test with a P value of 0.05 compared to cells propagated in growth medium.
