# Supplementary material for: In Vitro Effects of Papaverine on Cell Proliferation, Reactive Oxygen Species, and Cell Cycle Progression in Cancer Cells
Source: Molecules. 2021 Oct 22;26(21):6388. doi: 10.3390/molecules26216388 (PMC8587410; doi:10.3390/molecules26216388)
Supplement: Supplementary file 1 [file molecules-26-06388-s001.zip › molecules-1368532-supplementary/molecules-1368532-supplementary/Supplementary data/Supplementary 3.pdf]

Table S1: table displaying the effects of papaverine on oxidative stress as a change of fluorescence intensity relative to the fluorescence intensity of cells propagated in growth medium on MDA-MB-231 cells compared to A549- and DU145 cell lines at 48 h. Statistical significance is represented by an * when using the student *t*-test with a *P* value of 0.05 compared to cells propagated in growth medium

Cell line	Vehicle-treated cells	10 µM PPV-treated cells	50 µM PPV-treated cells	100 µM PPV-treated cells	150 µM PPV-treated cells	ESE-ol-treated cells
**MDA-MB-231**	1.00 ± 0.04	1.05 ± 0.07	0.91 ± 0.1	1.07 ± 0.08	1.11 ± 0.10	1.91 ± 0.04*
**A549**	0.95 ± 0.04	1.09 ± 0.08*	1.23 ± 0.06*	1.18 ± 0.06*	1.14 ± 0.04*	1.94 ± 0.04*
**DU 145**	1.00 ± 0.02*	0.92 ± 0.02*	0.90 ± 0.07	0.96 ± 0.04	0.99 ± 0.04	1.78 ± 0.06*

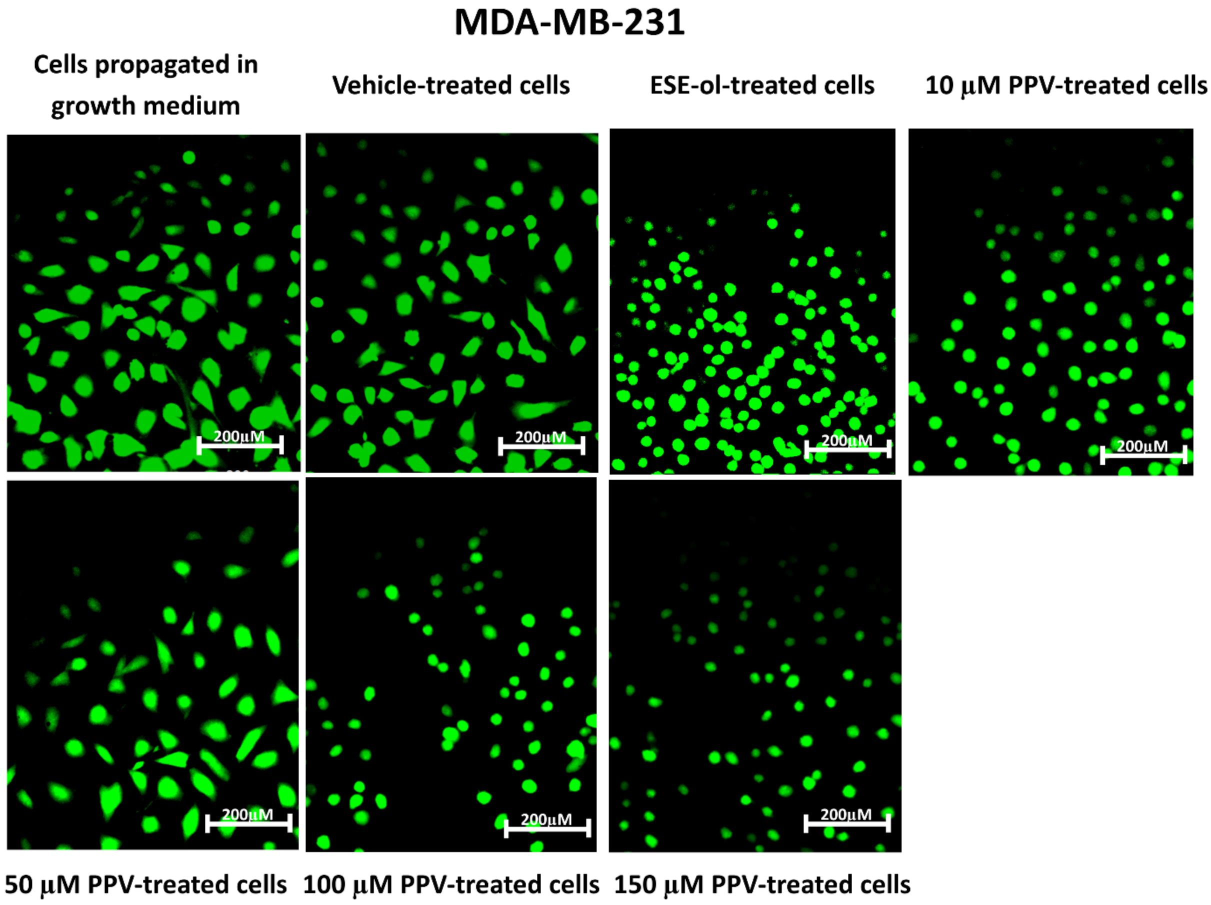

Figure S1. Fluorescence staining showing H_2O_2 production in MDA-MB-231 cells after 48 h. Fluorescence microscopy images of DCFDA staining demonstrating the effects of PPV (10-150 µM) on the fluorescent intensity on MDA-MB-231 cells at 48 h at a magnification of x20. A scale bar of 200µM is included.

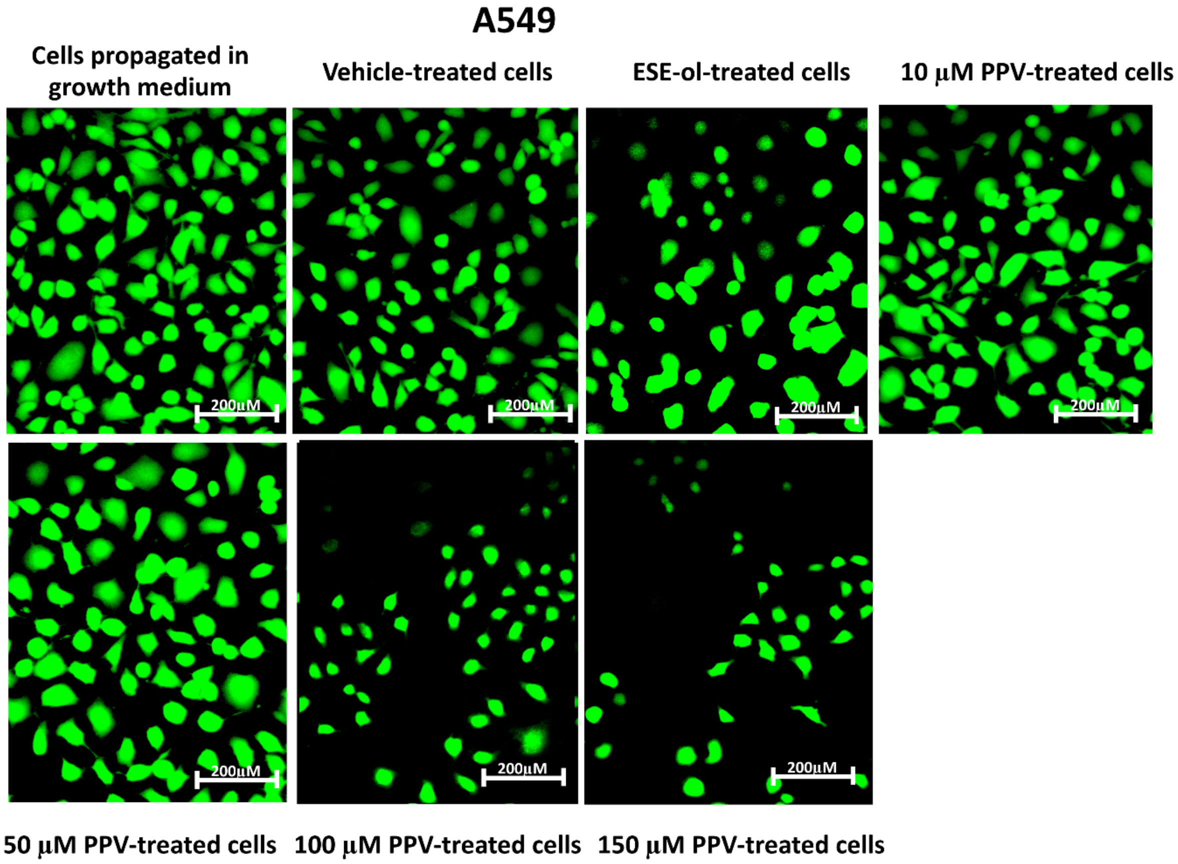

Figure S2. Fluorescence staining showing H_2O_2 production in A549 cells after 48 h. Fluorescence microscopy images of DCFDA staining demonstrating the effects of PPV (10-150 µM) on the fluorescent intensity on A549 cells at 48 h at a magnification of x20. A scale bar of 200µM is included.

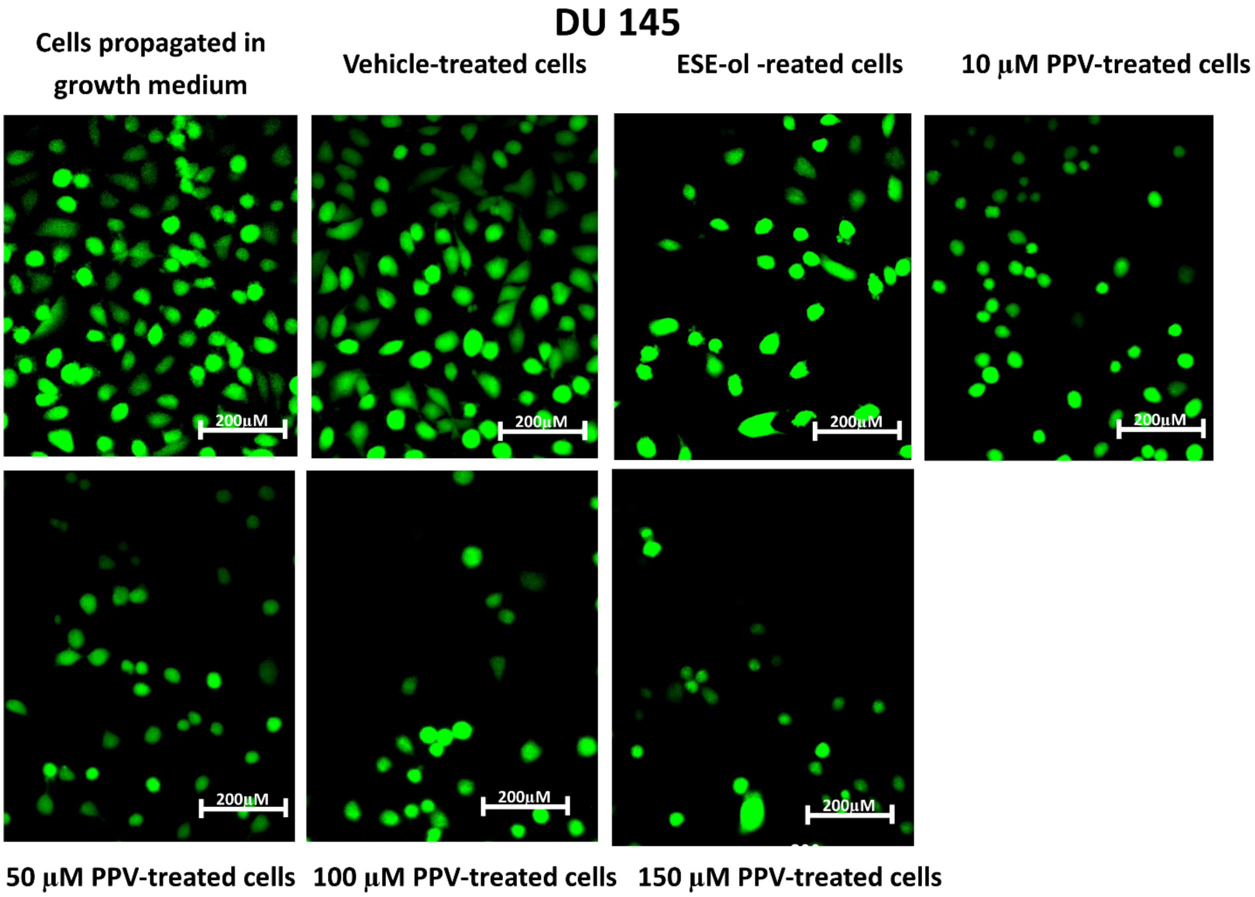

Figure S3. Fluorescence staining showing H_2O_2 production in DU145 cells after 48 h. Fluorescence microscopy images of DCFDA staining demonstrating the effects of PPV (10-150 µM) on the fluorescent intensity on DU145 cells at 48 h at a magnification of x20. A scale bar of 200µM is included.

Table S2: table displaying the effects of papaverine on oxidative stress as a change of fluorescence intensity relative to the fluorescence intensity of cells propagated in growth medium on MDA-MB-231 cells compared to A549- and DU145 cell lines at 72 h. Statistical significance is represented by an * when using the student *t*-test with a *P* value of 0.05 compared to cells propagated in growth medium.

Cell line	Vehicle-treated cells	10 µM PPV-treated cells	50 µM PPV-treated cells	100 µM PPV-treated cells	150 µM PPV-treated cells	ESE-ol-treated cells
**MDA-MB-231**	0.97 ± 0.00*	1.04 ± 0.04	0.73 ± 0.02*	0.83 ± 0.03*	0.84 ± 0.01*	1.95 ± 0.02*
**A549**	0.98 ± 0.02	1.02 ± 0.03	0.92 ± 0.03*	0.75 ± 0.04*	0.69 ± 0.03*	1.94 ± 0.03*
**DU 145**	0.99 ± 0.01	1.44 ± 0.02*	1.05 ± 0.05	1.14 ± 0.04*	1.15 ± 0.02*	1.83 ± 0.03*

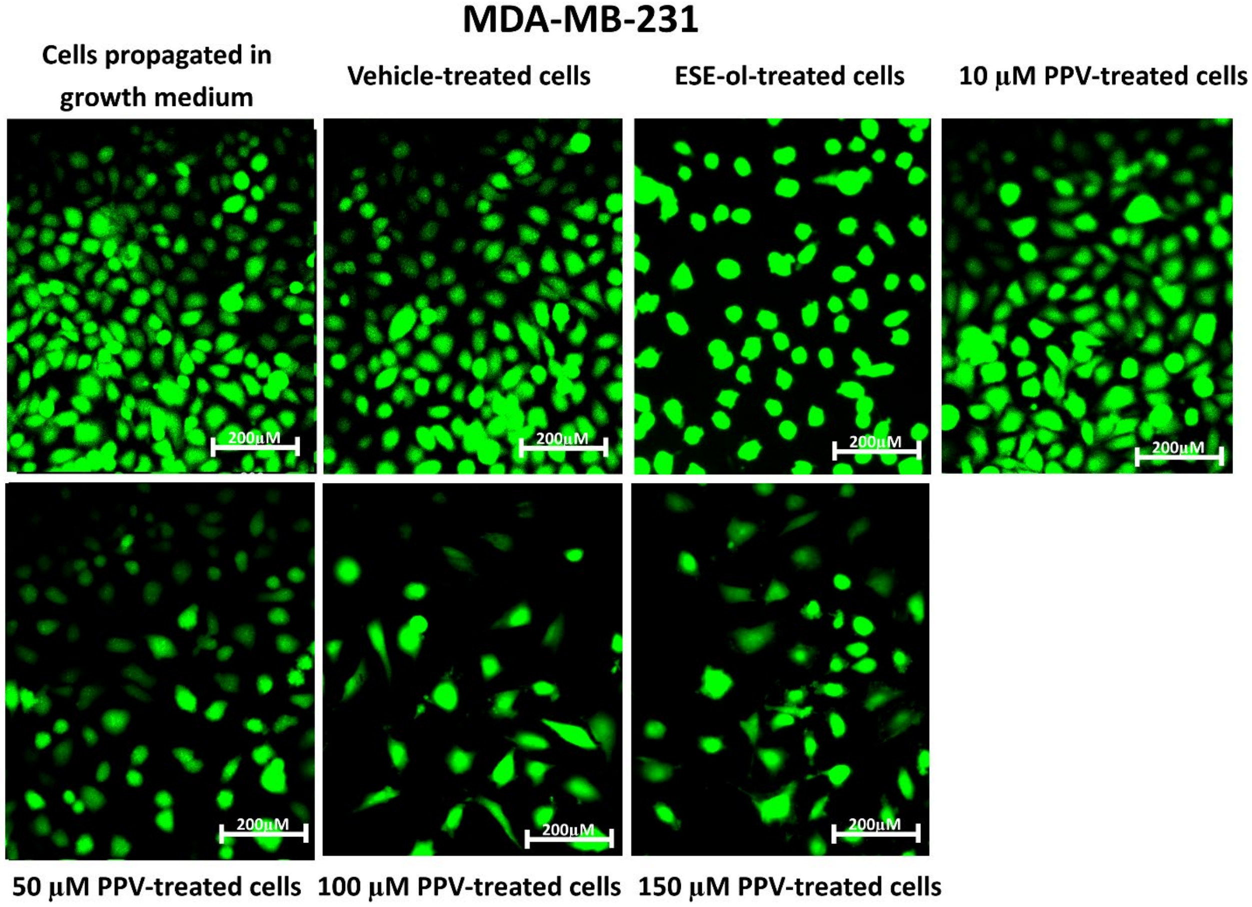

Figure S4. Fluorescence staining showing H_2O_2 production in MDA-MB-231 cells after 72 h. Fluorescence microscopy images of DCFDA staining demonstrating the effects of PPV (10-150 μM) on the fluorescent intensity on MDA-MB-231 cells at 72 h at a magnification of x20. A scale bar of 200μM is included.

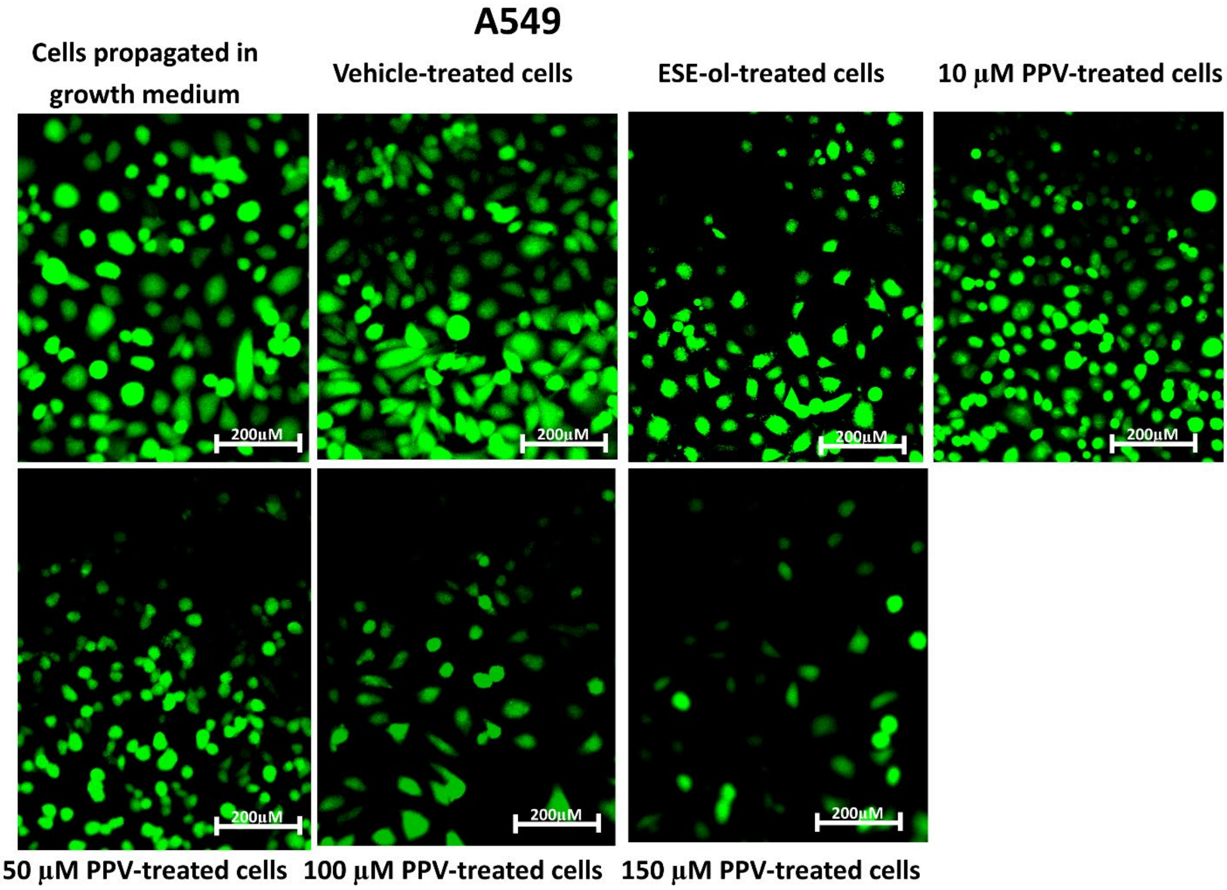

Figure S5. Fluorescence staining showing H_2O_2 production in A549 cells after 72 h. Fluorescence microscopy images of DCFDA staining demonstrating the effects of PPV (10-150 μM) on the fluorescent intensity on A549 cells at 72 h at a magnification of x20. A scale bar of 200μM is included.

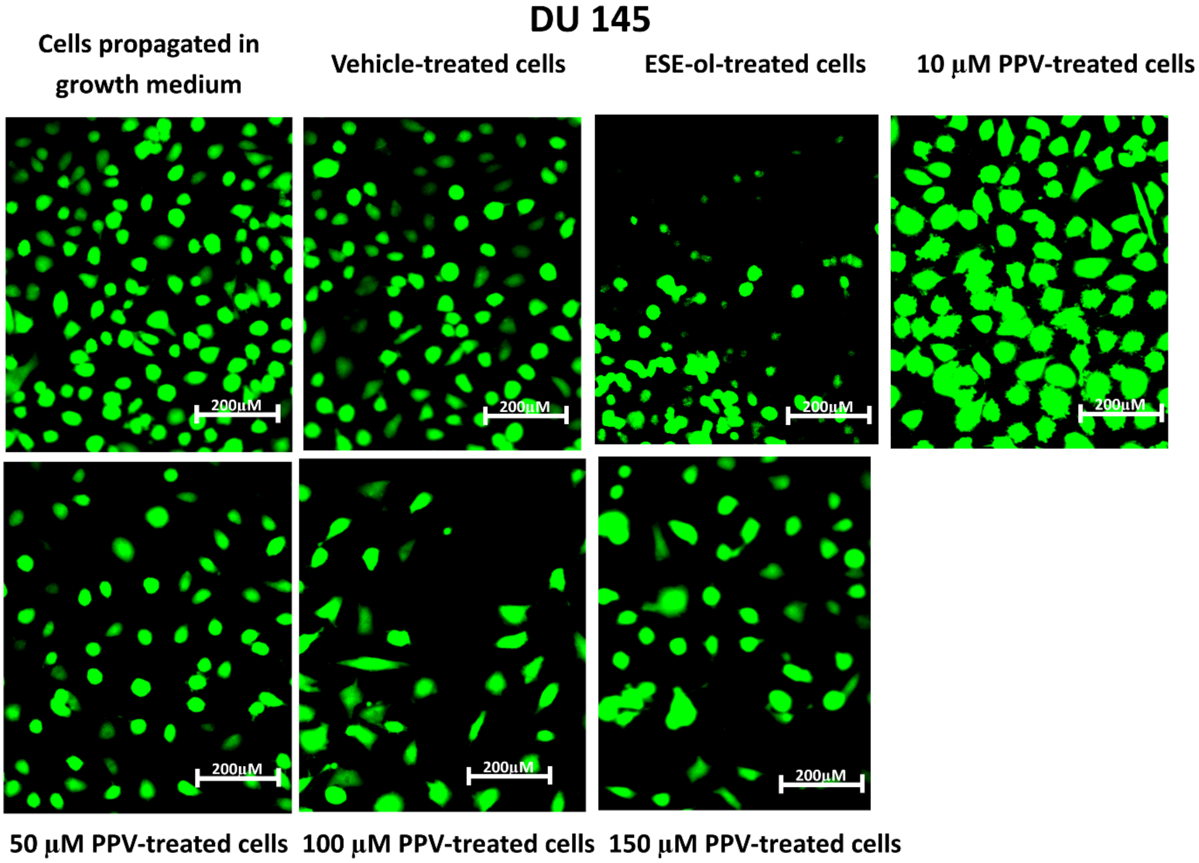

Figure S6. Fluorescence staining showing H_2O_2 production in DU145 cells after 72 h. Fluorescence microscopy images of DCFDA staining demonstrating the effects of PPV (10-150 µM) on the fluorescent intensity on DU145 cells at 72 h at a magnification of x20. A scale bar of 200µM is included.
