# Supplementary material for: In Vitro Effects of Papaverine on Cell Proliferation, Reactive Oxygen Species, and Cell Cycle Progression in Cancer Cells
Source: Molecules. 2021 Oct 22;26(21):6388. doi: 10.3390/molecules26216388 (PMC8587410; doi:10.3390/molecules26216388)
Supplement: Supplementary file 1 [file molecules-26-06388-s001.zip › molecules-1368532-supplementary/molecules-1368532-supplementary/Supplementary data/Supplementary 4.pdf]

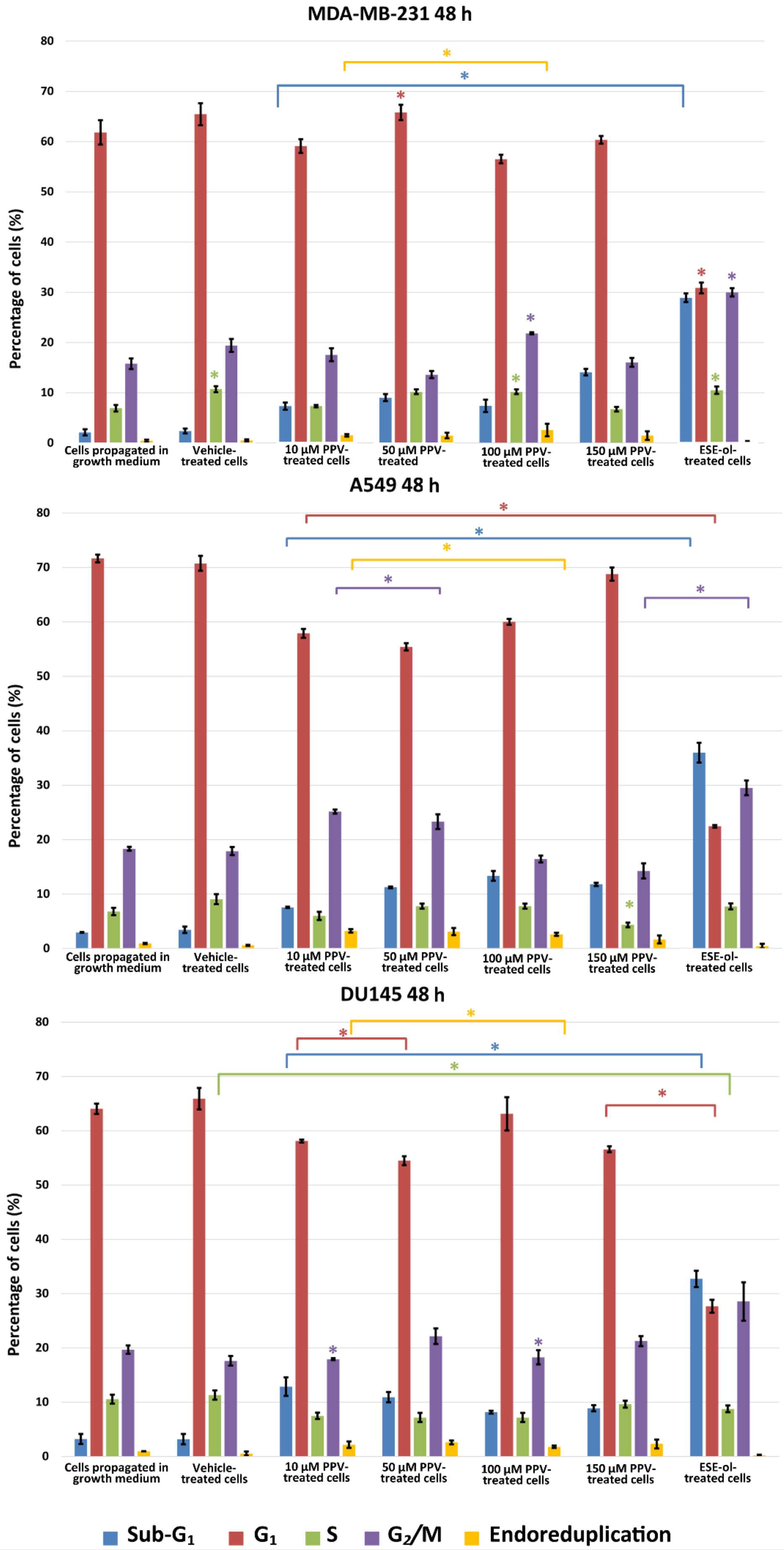

Supplementary figure S1: Flow cytometry results demonstrating the effects of PPV (10-150 µM) on the

cell cycle on MDA-MB-231-, A549- and DU145 cells at 48 h.

*The blue bar represents the percentage of cells in Sub-G_1 phase, the red bar represents the percentage of cells in G_1 phase, the green bar represents the percentage of cells in S phase, the purple bar represents the percentage of cells in G_2/M phase and the yellow bar represents the percentage of cells undergoing endoreduplication. Statistical significance is represented by an * when using the student t-test with a P value of 0.05 compared to cells propagated in growth medium.*

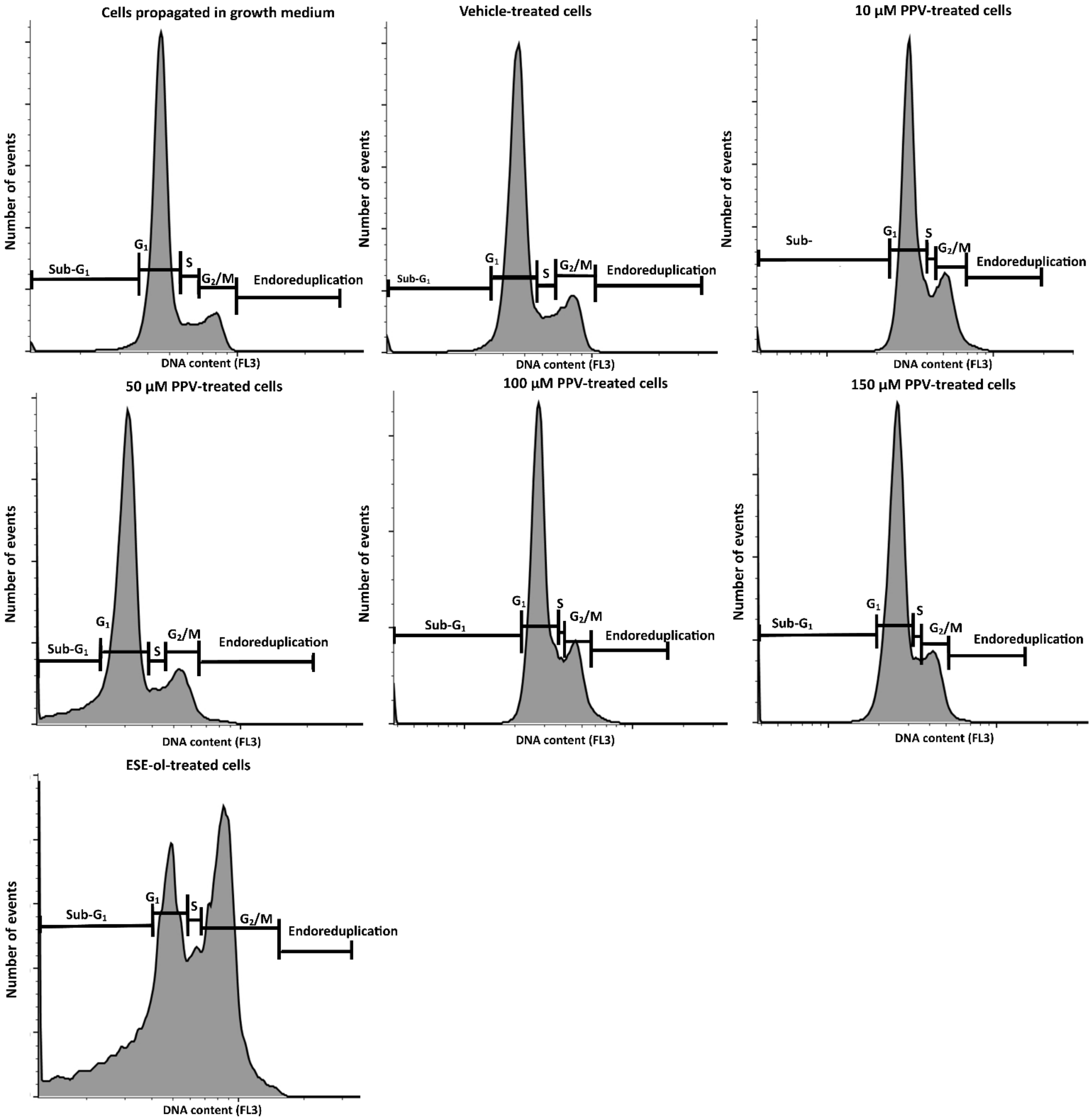

Supplementary figure S2: Cell cycle progression of MDA-MB-231 cells treated with PPV (10-150 µM) at 48 h.

PPV-treated cells showed an increase in the number of cells in the sub-G_1 phase with an increase in concentration. ESE-ol-treated cells were used as a positive control and resulted in an increase in cell number in the sub-G_1 and G_2/M phase.

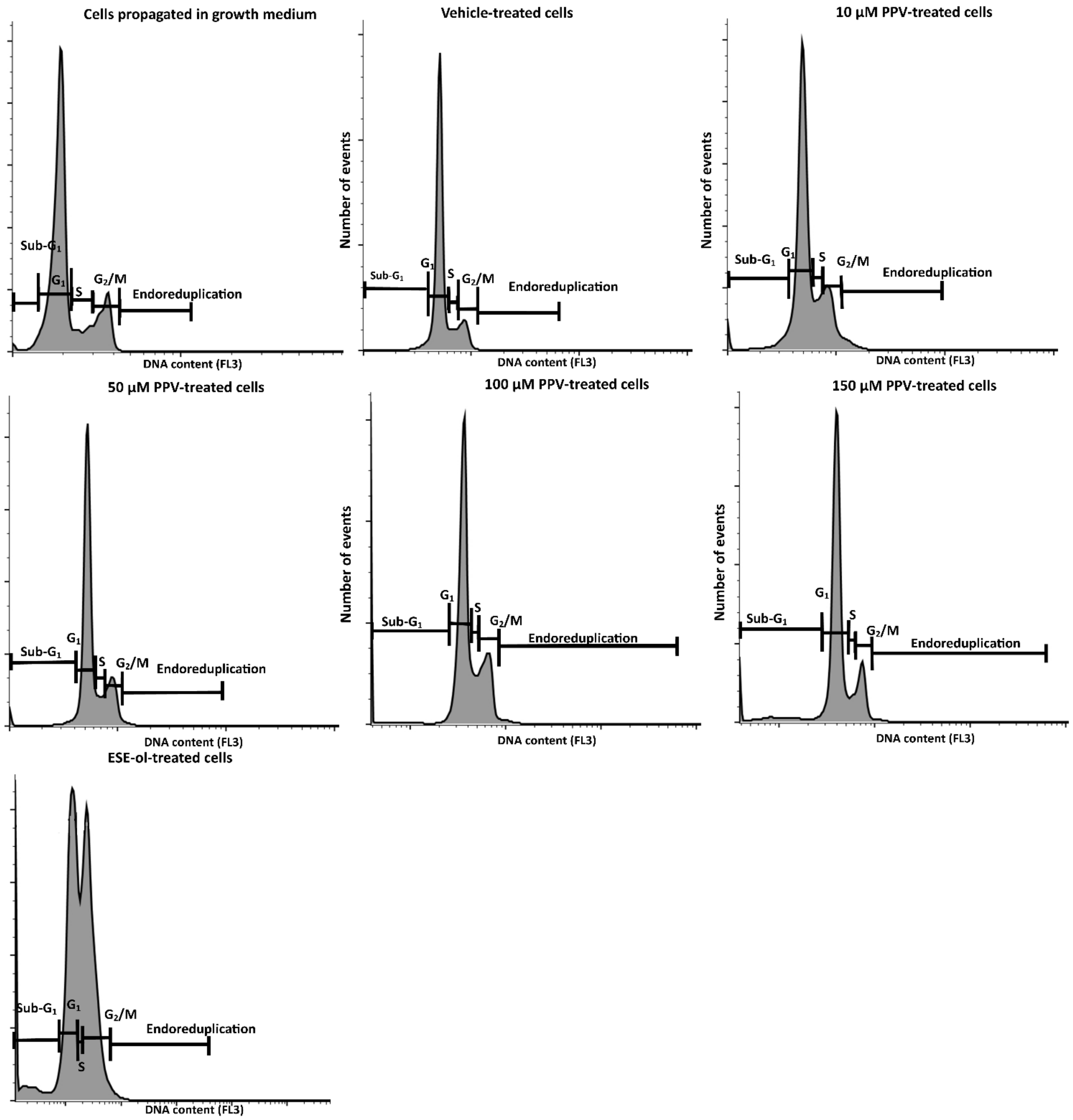

Supplementary figure S3: Cell cycle progression of A549 cells treated with PPV (10-150 µM) at 48 h.

PPV-treated cells showed an increase in the number of cells in the sub-G_1 phase with an increase in concentration. ESE-ol-treated cells were used as a positive control and resulted in an increase in cell number in the sub-G_1 and G_2/M phase.

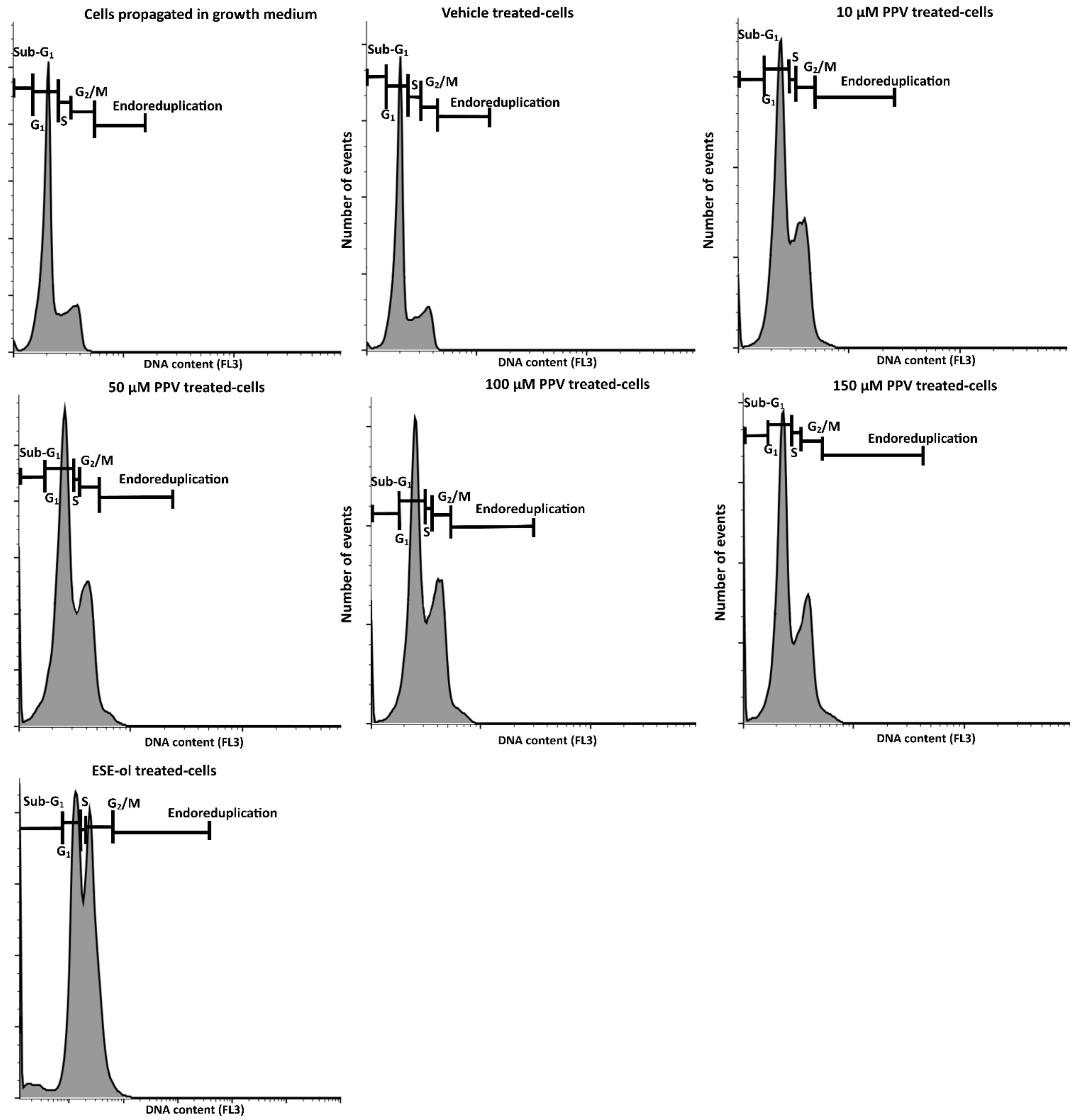

Supplementary figure S4: Cell cycle progression of DU145 cells treated with PPV (10-150 µM) at 48 h.

PPV-treated cells showed an increase in the number of cells in the sub-G_1 phase with an increase in concentration. ESE-ol-treated cells were used as a positive control and resulted in an increase in cell number in the sub-G_1 and G_2/M phase.

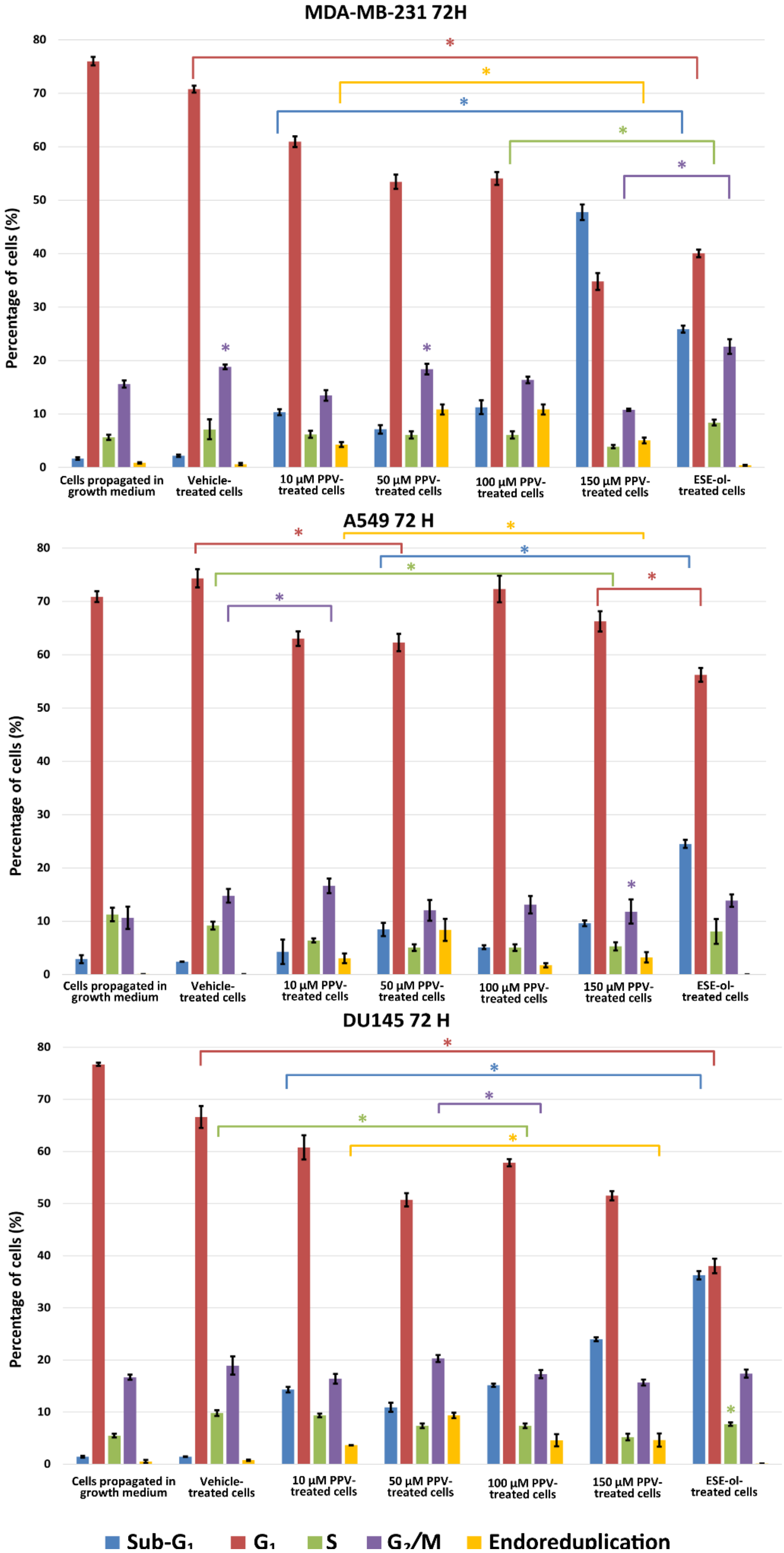

Supplementary figure S5: Flow cytometry results demonstrating the effects of PPV (10-150 µM) on the cell cycle on MDA-MB-231-, A549- and DU145 cells at 72 h.

*The blue bar represents the percentage of cells in Sub-G1 phase, the red bar represents the percentage of cells in G1 phase, the green bar represents the percentage of cells in S phase, the purple bar represents the percentage of cells in G2/M phase and the yellow bar represents the percentage of cells undergoing endoreduplication. Statistical significance is represented by an * when using the student t-test with a P value of 0.05 compared to cells propagated in growth medium*

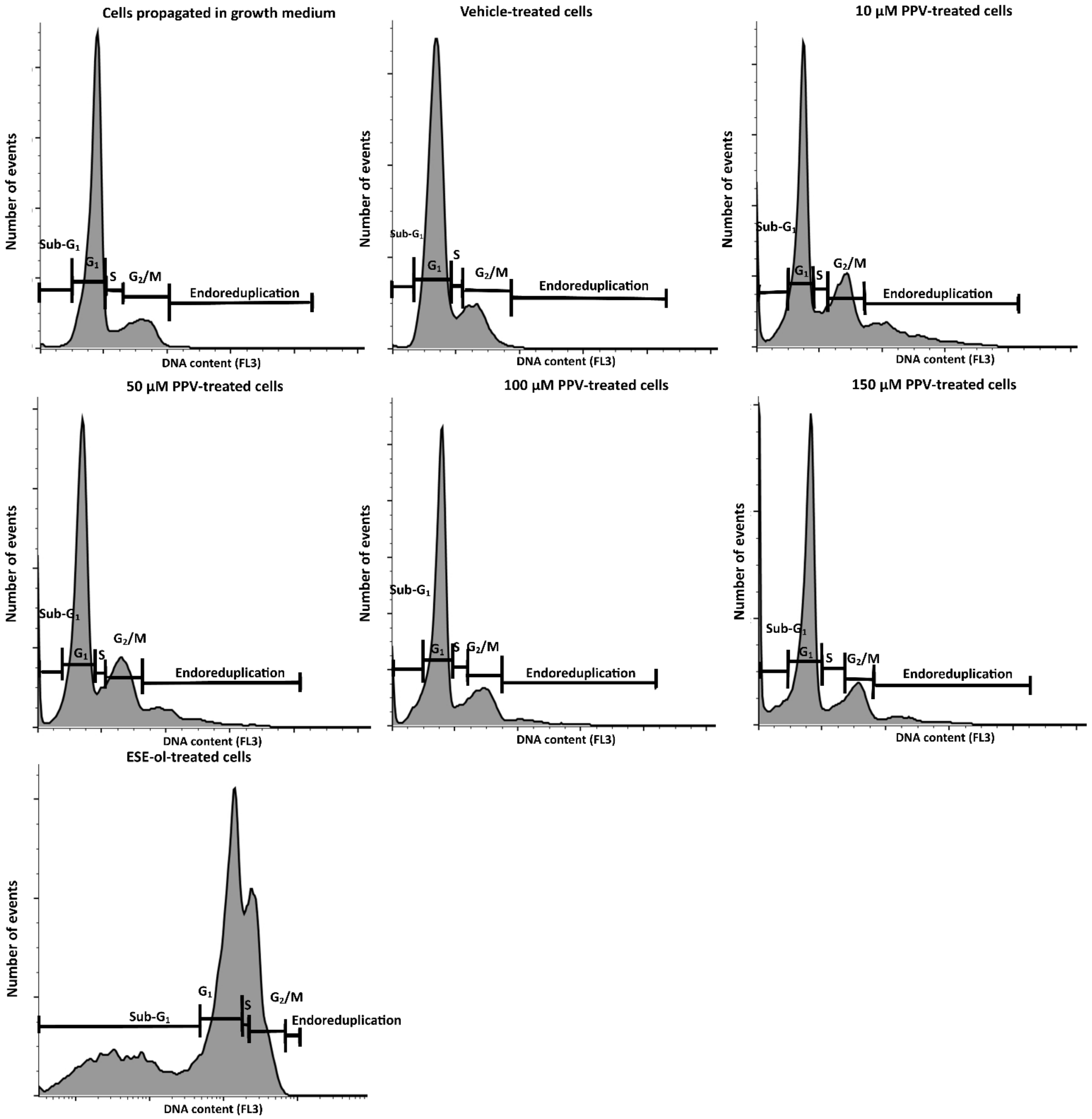

Supplementary figure S6: Cell cycle progression of MDA-MB-231 cells treated with PPV (10-150 µM) at 72 h.

PPV-treated cells showed an increase in the number of cells in the sub-G_1 phase with an increase in concentration. ESE-ol-treated cells were used as a positive control and resulted in an increase in cell number in the sub-G_1 and G_2/M phase.

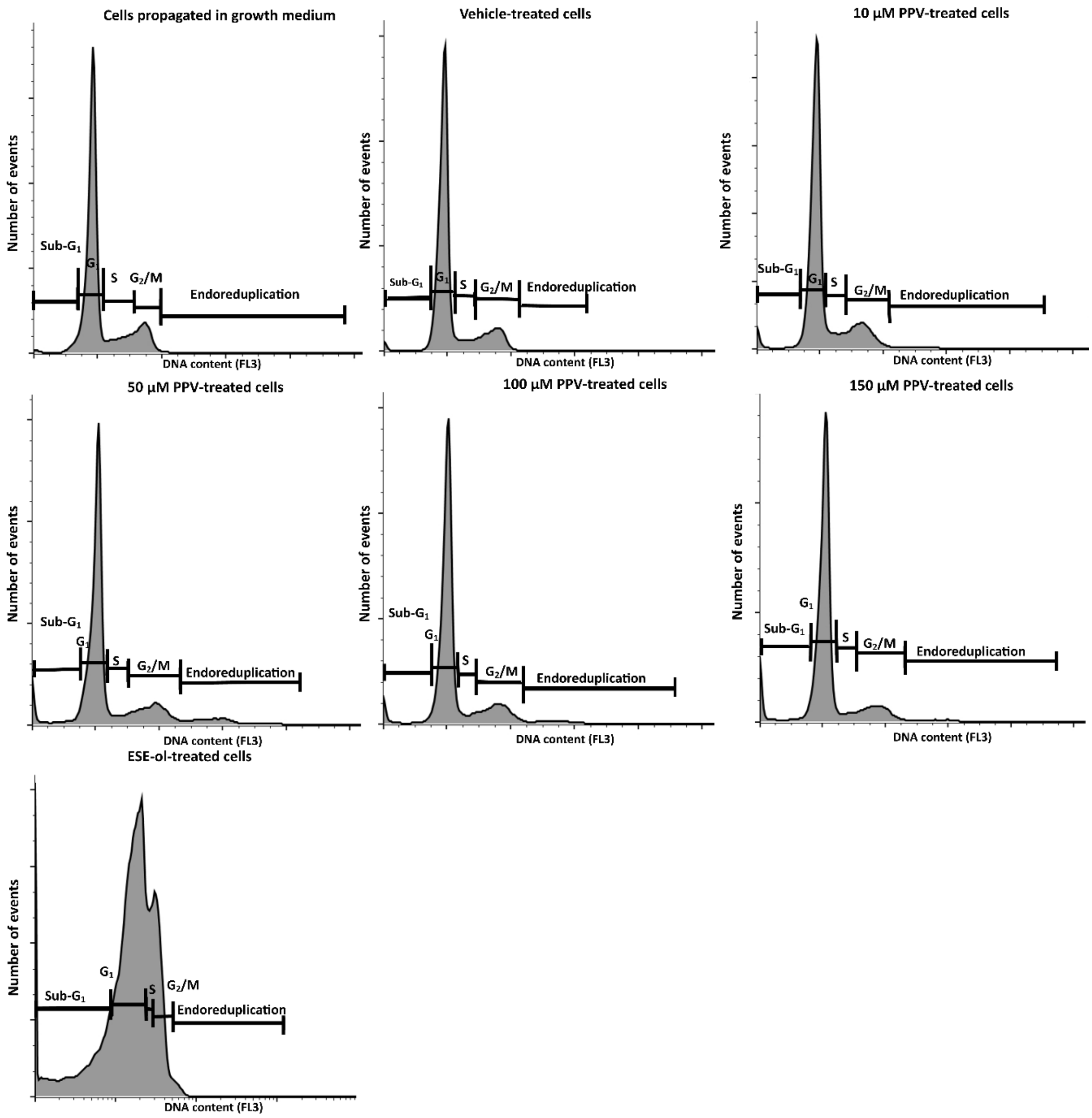

Supplementary figure S7: Cell cycle progression of A549 cells treated with PPV (10-150 μM) at 72 h.

PPV-treated cells showed an increase in the number of cells in the sub-G_1 phase with an increase in concentration. ESE-ol-treated cells were used as a positive control and resulted in an increase in cell number in the sub-G_1 and G_2/M phase.

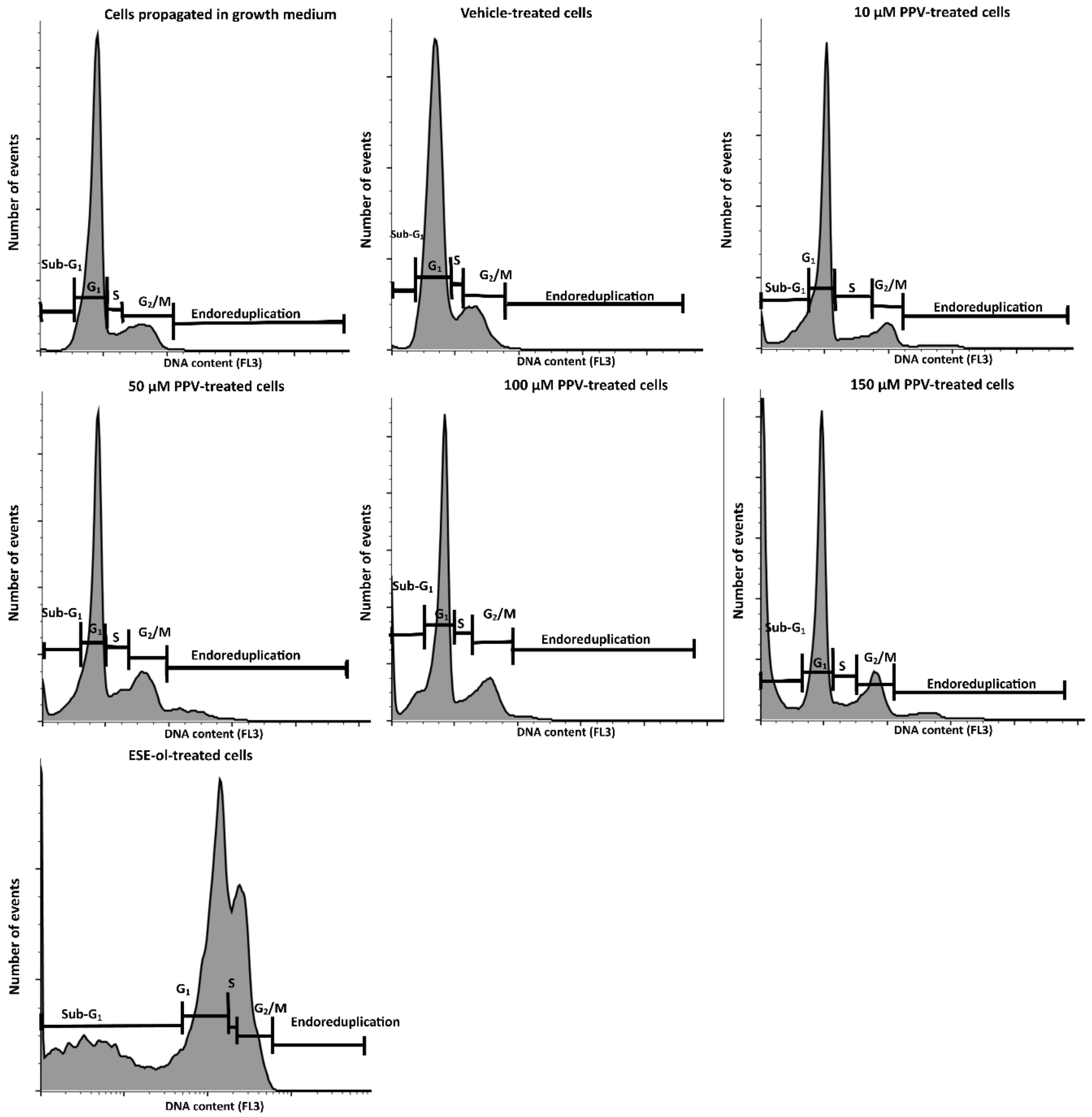

Supplementary figure S8: Cell cycle progression of DU145 cells treated with PPV (10-150 µM) at 72 h.

PPV-treated cells showed an increase in the number of cells in the sub-G_1 phase with an increase in concentration. ESE-ol-treated cells were used as a positive control and resulted in an increase in cell number in the sub-G_1 and G_2/M phase.
